# Structural Basis for Dityrosine-Mediated Inhibition of α-Synuclein Fibrillization

**DOI:** 10.1021/jacs.2c03607

**Published:** 2022-06-24

**Authors:** Cagla Sahin, Eva Christina Østerlund, Nicklas Österlund, Joana Costeira-Paulo, Jannik Nedergaard Pedersen, Gunna Christiansen, Janni Nielsen, Anne Louise Grønnemose, Søren Kirk Amstrup, Manish K. Tiwari, R. Shyama Prasad Rao, Morten Jannik Bjerrum, Leopold L. Ilag, Michael J. Davies, Erik G. Marklund, Jan Skov Pedersen, Michael Landreh, Ian Max Møller, Thomas J. D. Jørgensen, Daniel Erik Otzen

**Affiliations:** α Interdisciplinary Nanoscience Center (iNANO), Aarhus University, Gustav Wieds Vej 14, DK-8000 Aarhus C, Denmark; β Department of Molecular Biology and Genetics, Aarhus University, Universitetsbyen 81, DK-8000 Aarhus C, Denmark; χ Department of Biochemistry and Molecular Biology, University of Southern Denmark, Campusvej 55, DK-5230 Odense M, Denmark; δ Department of Biochemistry and Biophysics, Stockholm University, SE-114 18 Stockholm, Sweden; ε Department of Chemistry - BMC, BMC − Uppsala University, Box 576, SE-751 23 Uppsala, Sweden; ϕ Department of Health Science and Technology, Medical Microbiology and Immunology, Aalborg University, Fredrik Bajers Vej 3b, DK-9220 Aalborg Ø, Denmark; γ Department Chemistry, University of Copenhagen, Universitetsparken 5, DK-2100 Copenhagen Ø, Denmark; η Biostatistics and Bioinformatics Division, Yenepoya Research Center, Yenepoya University, Mangaluru-575018, Karnataka, India; ι Department of Materials and Environmental Chemistry, Stockholm University, SE-114 18 Stockholm, Sweden; j Department of Biomedical Sciences, University of Copenhagen, Blegdamsvej 3B, DK-2200 Copenhagen N, Denmark; κ Department of Chemistry, Aarhus University, Gustav Wieds Vej 14, DK-8000 Aarhus C, Denmark; λ Department of Microbiology, Tumor and Cell Biology, Karolinska Institutet, Solnavägen 9, SE-171 65 Solna, Sweden; μ Department of Molecular Biology and Genetics, Aarhus University, Forsøgsvej 1, DK-4200 Slagelse, Denmark

## Abstract

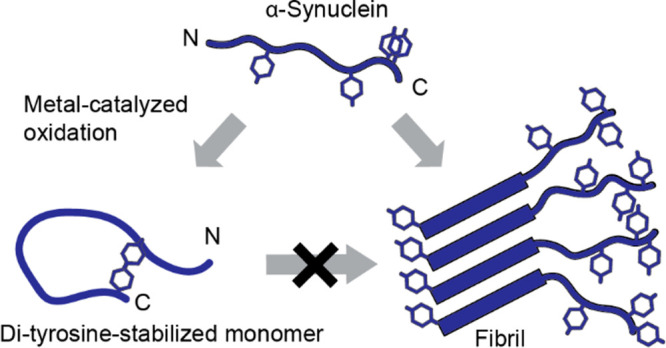

α-Synuclein (α-Syn) is an intrinsically disordered protein which self-assembles into highly organized β-sheet structures that accumulate in plaques in brains of Parkinson’s disease patients. Oxidative stress influences α-Syn structure and self-assembly; however, the basis for this remains unclear. Here we characterize the chemical and physical effects of mild oxidation on monomeric α-Syn and its aggregation. Using a combination of biophysical methods, small-angle X-ray scattering, and native ion mobility mass spectrometry, we find that oxidation leads to formation of intramolecular dityrosine cross-linkages and a compaction of the α-Syn monomer by a factor of √2. Oxidation-induced compaction is shown to inhibit ordered self-assembly and amyloid formation by steric hindrance, suggesting an important role of mild oxidation in preventing amyloid formation.

α-Synuclein (α-Syn) is a 140-residue intrinsically disordered protein whose exact physiological role remains unknown.^1^ However, it is strongly associated with Parkinson’s disease (PD) and forms inclusions known as Lewy bodies in the brains of PD patients.^2^ Metal-ion-catalyzed oxidation (MCO) is believed to play a significant role in the origin and progression of PD.^3,4^ However, common MCO modifications, including carbonylation of the side chains of Lys, Pro, Arg, and Thr residues, occur only to a low extent with α-Syn.^5^ MCO of α-Syn predominantly leads to the oxidation of Met to sulfoxides^5,6^ and the formation of dityrosine (diTyr) linkages.^5^ These modifications favor assembly into soluble aggregates rather than fibrils.^7−9^ DiTyr cross-linkages, both intra- and intermolecular, are associated with oxidative stress^8,10−12^ and have been identified *post mortem* in brains of PD patients.^13^ Intermolecular diTyr formation connecting Tyr39-Tyr39 results in covalent α-Syn dimers that have been shown to have various effects on α-Syn aggregation.^10,12,13^ At early time points of α-Syn MCO, the formation of intramolecular diTyr cross-linked α-Syn monomers was favored over the formation of diTyr-linked dimers.^5^ This raises the question of how early oxidative modifications influence the fibrillization mechanism.

To answer this question, we employed an MCO protocol^5^ combining Cu^2+^ and H_2_O_2_ to investigate how early α-Syn modifications, mainly Met oxidations and diTyr cross-links, affect structure and amyloidogenic properties.

α-Syn contains four Tyr residues, one in the N-terminal region (Tyr39) and three in close proximity in the C-terminal tail (Tyr125, Tyr133, and Tyr136) (Figure 1A). Monitoring Tyr and diTyr fluorescence in parallel, it is seen that diTyr is formed rapidly in α-Syn upon MCO with a half-life of 0.86 min, in reasonable accord with a half-life of 1.41 min for Tyr fluorescence decay (Figure 1B). We used mass spectrometry (MS) to characterize modifications within the intact protein. MCO of α-Syn showed up to three +16 Da increases for 15 min of oxidation (Figure S1A) and more for longer incubation times. LC-MS/MS supported the presence of Met sulfoxides and sulfones (data not shown). The deconvoluted mass spectrum further showed a −2 Da loss for the oxidized wild-type (wt) protein (Figure 1C and Figure S1B), corresponding to an intramolecular Tyr cross-link formation (loss of 2 H). Tyr→Phe mutations in either the N-terminal (Tyr39Phe) or in the C-terminal tail (Tyr125/133/136Phe) of α-Syn led to similar modifications, i.e. Met oxidations, as well as loss of 2 H, suggesting MCO-induced cross-links. However, since Tyr125/133/136Phe α-Syn contains only one Tyr residue, other cross-links can be formed, e.g., between Tyr39 and one of the 15 Lys residues^14^ or His50. A shift in charge state distribution toward lower charge states for wt and the Tyr125/133/136Phe variant (Figure S1C) indicates a shift toward compact conformations.^15^ The rate of Met oxidation was not affected by the Tyr→Phe mutations using N15-αSyn (Figure S2).

**Figure 1:**
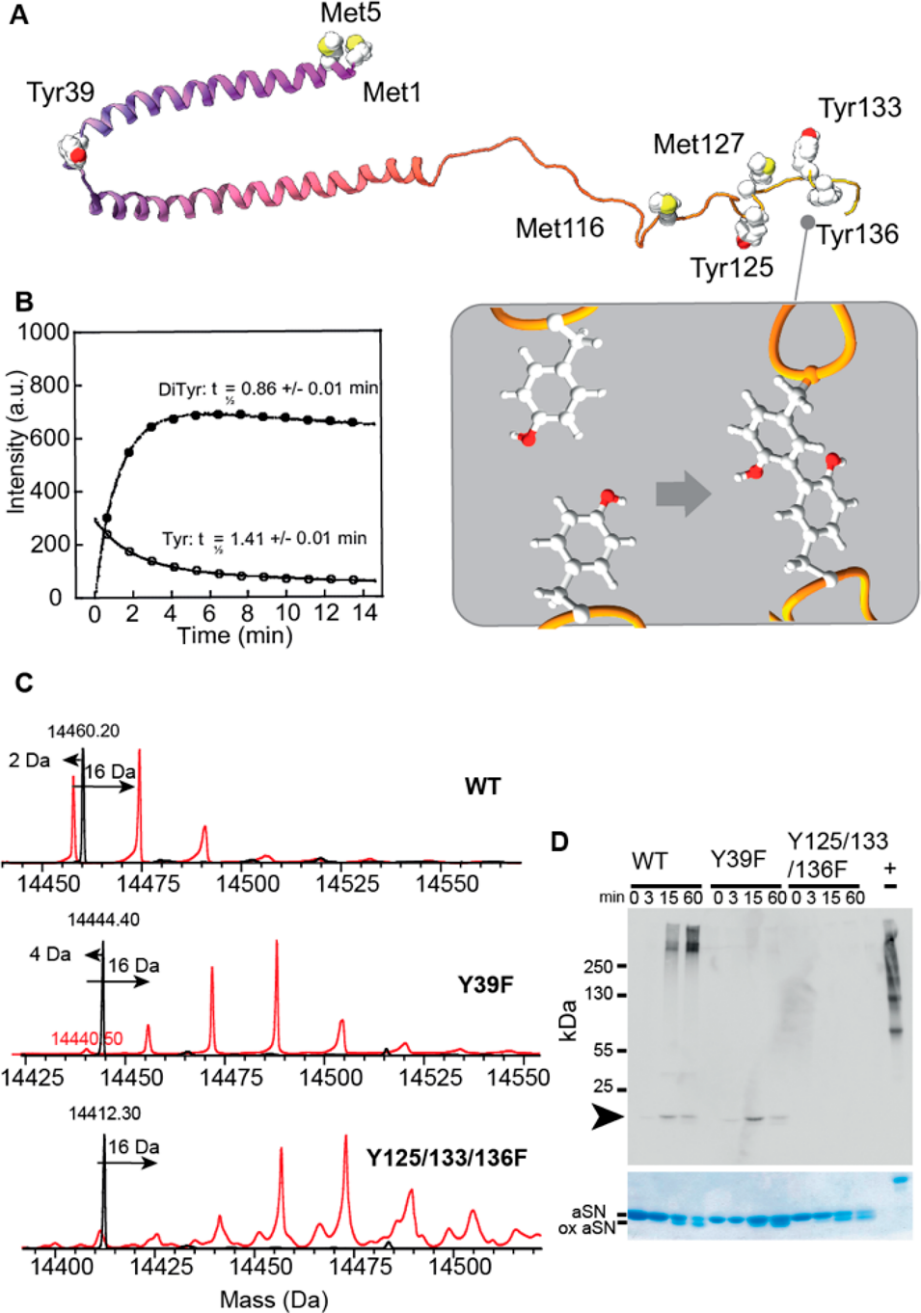
DiTyr formation of α-Syn. (A) Schematic of α-Syn (PDB: 1XQ8, SDS micelle bound). Possible oxidation sites indicated. Insert: diTyr formation. (B) Time course of Tyr and diTyr fluorescence for wt α-Syn under oxidative conditions. Data fitted to a single-exponential decay with linear drift. Fit shown with a solid line. (C) Deconvoluted MS spectra of wt, Tyr39Phe, and Tyr125/133/136Phe α-Syn either unmodified (black) or 15 min oxidized (red). Arrows showing decrease and increase of molecular mass. (D) Top: DiTyr detection on an immunoblot of wt, Tyr39Phe, and Tyr125/133/136Phe α-Syn, oxidized for 0, 3, 15, and 60 min. Monomeric diTyr is indicated by an arrowhead. Positive control: oxidized α-casein. Bottom: Coomassie-stained SDS-PAGE showing different migration patterns of unmodified and oxidized α-Syn.

To test how different Tyr configurations affect aggregation, we followed MCO of α-Syn wt, Tyr39Phe, and Tyr125/133/136Phe using immunoblotting with a diTyr-specific monoclonal antibody. After 3 min, bands indicating diTyr formation were detected, with an increase in intensity at 15 min for both the wt and the Tyr39Phe samples. SDS-PAGE analysis showed formation of faster-migrating species (Figure 1D, bottom, and Figure S4). Higher molecular weight bands detected for wt suggest cross-linked oligomers. As expected, no diTyr was detected in the triple mutant (Figure 1D top). The loss of 2 Da supports formation of an intramolecular cross-link (Figures S2 and S3). The immunoblot highlights the importance of diTyr cross-links in the oxidation of α-Syn but does not rule out alternative cross-links formed in parallel.

We then asked whether these intramolecular diTyr links affect the conformational preferences of α-Syn. Small-angle X-ray scattering (SAXS) data for unoxidized and 60 min oxidized α-Syn display Guinier behavior, i.e., a relatively constant level at low values of the modulus of the scattering vector, *q*, followed by a power-law behavior (linear decline in the log–log plot) at intermediate *q*, characteristic of polymer-like structures (Figure 2). Radii of gyration (*R*_g_) from indirect Fourier transformation (IFT, see the Supporting Information (SI)) are given in Table 1. Native α-Syn showed an *R*_g_ of 3.96 nm, in good agreement with an extended α-Syn conformation^16,17^ (Figure 2, Table 1, S5). *R*_g_ was reduced to 2.69 nm (a factor 1.47) upon MCO. An ideal ring (joined at the two termini) will have an *R*_g_ that is √2 (∼1.4) smaller than that of an ideal chain (see SI), suggesting that MCO induced a conversion from disordered to compact monomer.

**Figure 2:**
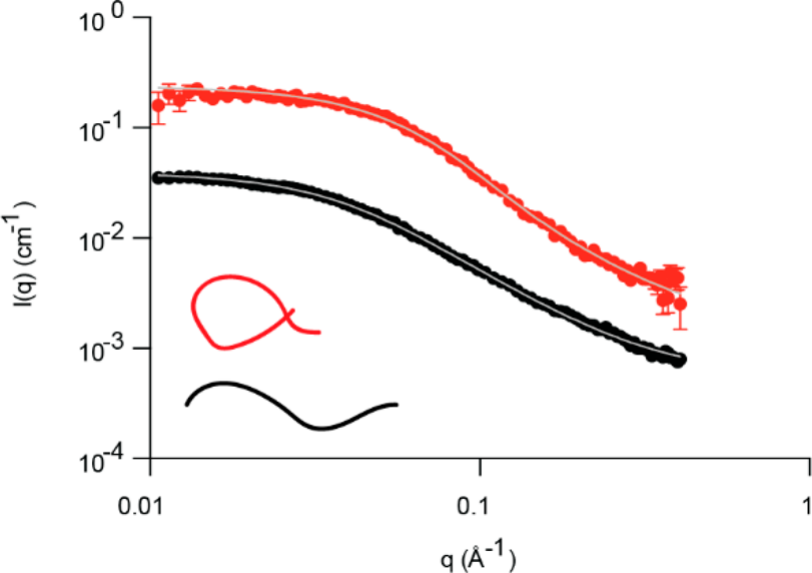
SAXS scattering curves for unmodified (black; without scale factor) and oxidized monomer (red; with a scale factor of 10). The connectivity of the models used for fitting the data is shown schematically but not to scale (top: loop-containing model, bottom: linear chain model). Fits are solid lines.

**Table 1:** Results from SAXS Analysis^a^

	*c* [mg/mL]	*R*_g_(IFT) [nm]	*c*(model) [mg/mL]	*R*_g_(model) [nm]	*b*(model) [nm]	χ^2^(model)
monomer	4.0	3.96 ± 0.02	4.00 ± 0.03	3.80	1 71 ± 0.02	1.0
oxidized monomer	2.2	2.69 ± 0.02	2.55 ± 0.02	2.95	1.96 ± 0.03	1.1

a *c*, concentration measured by absorbance; *R*_g_(IFT), radius of gyration from IFT; *c*(model), concentration determined from the model fits (linear chain model for native monomer and ring model for oxidized monomer); *R*_g_(model), radius of gyration of the two models determined numerically from the low-*q* range of the model curves; *b*, Kuhn length; χ^2^, reduced weighted chi-square.

The scattering curves were subsequently fitted by the models derived in the SI (fits in Figure 2, summarized in Table 1). The native monomer is in good agreement with the linear chain model, giving a concentration value identical to the one determined by absorbance measurements and a Kuhn length^18^ only slightly larger than the expected value of 1.51 nm.^19^ The oxidized monomer is best described by a loop-containing model, which represents a structure with a link from Tyr39 to one of the three C-terminal Tyr residues.

Based on the SAXS data, we hypothesize that MCO could promote α-Syn compaction through intramolecular diTyr formation. To investigate this, we used native ion mobility mass spectrometry (IM-MS) (Figure 3A). Briefly, from the time it takes ions to traverse a gas-filled drift cell, we can calculate their collision cross sections (CCSs), giving information on their conformational preferences. Analysis of unmodified α-Syn revealed a CCS distribution centered around 2400 Å^2^ for all major charge states (Figure 3B). Five minutes of MCO did not notably increase dimers (cf. Figure 1) but shifted the CCS of the monomers toward a compact state with CCS ≈ 1900 Å^2^ for lower charge states (Figure 3B), consistent with our extended-to-ring-transformation hypothesis. A direct correlation between molecular weight and CCS is observed (Figure S6).

**Figure 3:**
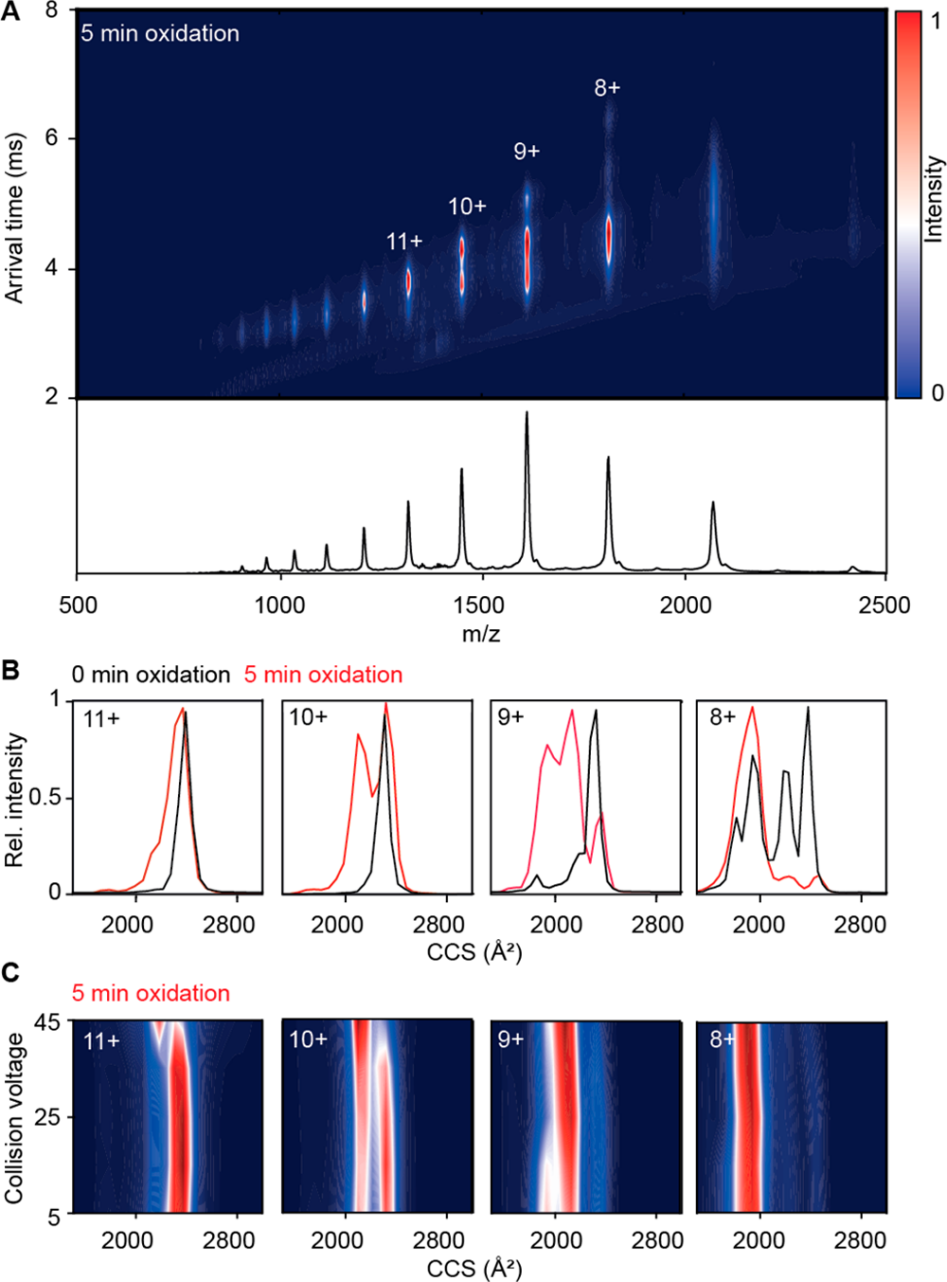
IM-MS analysis of oxidized α-Syn. (A) Spectrum of 5 min oxidized wt α-Syn and ion mobiligrams showing +11 to +8 charge states. (B) Overlay of CCSs of wt untreated α-Syn (black) and oxidized wt α-Syn (red). (C) CIU shown from 5 to 45 V for the same charge states as given in the CCS plots (B).

To investigate the conformational stability of different α-Syn populations, we employed collision-induced unfolding (CIU). Here, the protein ions are subjected to increasing collisional activation in the ion trap of the mass spectrometer. The resulting change in CCS informs about the conformational stability of the ion.^20^ Interestingly, oxidized α-Syn showed no significant increase in CCS as the collisional activation was increased from 5 to 50 V, at which protein fragmentation (not unfolding) occurred (Figure 3C). The high resistance of the compact states to unfolding indicates covalent stabilization rather than altered non-covalent interactions in the oxidized monomer.

To corroborate that the compaction stems from intramolecular cross-links, we performed IM-MS of the Tyr39Phe single mutant and the Tyr125/133/136Phe triple mutant. To exclude effects from altered solution conformations in response to the Tyr-to-Phe mutations, we performed IM-MS under denaturing conditions using 50% acetonitrile with 0.1% formic acid (Figure S7). Under these conditions, all variants exhibited the same extended conformation as native wt monomer (Figure S7, black line). Following MCO, the denatured wt monomer underwent the same extended-to-compact shift as seen under native conditions. However, the oxidized Tyr39Phe mutant showed two populations with similar intensities, one more compact and the other extended. We speculate that, in this variant, the central His50 could be linked to a C-terminal Tyr, resulting in a smaller compact population. His50’s role as a Cu^2+^ ligand^21^ makes it an active site of oxidation. The oxidized triple mutant showed no pronounced shift to a compact conformation (Figure S7). This implicates C-terminal Tyr residues in compaction. IM-MS results suggest that diTyr formation involving the C-terminal and most likely Tyr39 leads to a stable, compact monomer. MS and SAXS on MCO-α-Syn reveal an intramolecular diTyr cross-link mainly between Tyr39 and a C-terminal Tyr, resulting in a compact conformation. Importantly, Tyr39 is located in the middle of the hydrophobic amyloid core of α-Syn fibrils.^22−25^ Mutating this Tyr partially prevents the generation of a covalently linked, compact monomer. This core region, together with a part of the amphiphilic N-terminus of α-Syn, forms part of the core of amyloid fibrils, whereas the C-terminus (residues 95–140 containing the remaining three Tyr) remains flexible and outside the cross-β-sheet structure,^23,26^ also upon Tyr39 phosphorylation.^27^ We therefore hypothesize that the steric hindrance imparted by an intramolecular cross-link from the amyloid core region to the C-terminus would inhibit fibril formation.

To test this, we used the amyloid-specific fluorophore thioflavin T (ThT) to follow fibrillization of α-Syn oxidized to various extents (Figure 4A). Indeed, the fibrillization lag time increased significantly from ∼8 to ∼30 h after 0.1 min of MCO. After 5 min of oxidation, no significant fluorescence signal was measured, indicating that fibrils were not formed. The fluorescence intensity does not *per se* rule out formation of other aggregates, but quantification of soluble species with SDS-PAGE confirmed that 5 min of oxidation resulted in completely soluble protein at the ThT assay end-point (Figure S8). Prolonged MCO formed diTyr-linked oligomers (Figure 1D).^28^ In contrast to other types of cross-linked oligomers,^29^ diTyr-linked oligomers are largely disordered.^30^ We used Fourier transformed infrared (FTIR) spectroscopy and circular dichroism (CD) to confirm the lack of fibrils in the end-point samples at 60 min of oxidation (Figure 4B,C). A peak at 1624 cm^–1^ in the deconvoluted FTIR spectrum indicates β-sheet structure as seen for unmodified α-Syn fibrils. This signal was significantly decreased for the oxidized samples. The CD absorbance in the far-UV region shows that oxidized α-Syn (monomer and end-point) lacks a persistent secondary structure, similar to the unstructured, unmodified α-Syn monomer, whereas β-sheet structure is observed for end-point-unmodified α-Syn, as expected for amyloid fibrils. Transmission electron microscopy (TEM) images showed fibrils formed under non-oxidized conditions, whereas fibril formation is largely inhibited during MCO, consistent with Met oxidation^31^ and our hypothesis that diTyr cross-links in the amyloid core of α-Syn prevent amyloid formation (Figure 4D). Since interactions in the β-strand-forming region (residues 35–96) are crucial for fibrillization, their disruption thus inhibits amyloid formation using Tyr residues specific to α-Syn (Figures S9 and S10).^25,32^ Our study shows how oxidation at the monomeric level prevents ordered aggregation, rather than oxidation of pre-existing assemblies, thereby switching between protective or pathological states.

**Figure 4:**
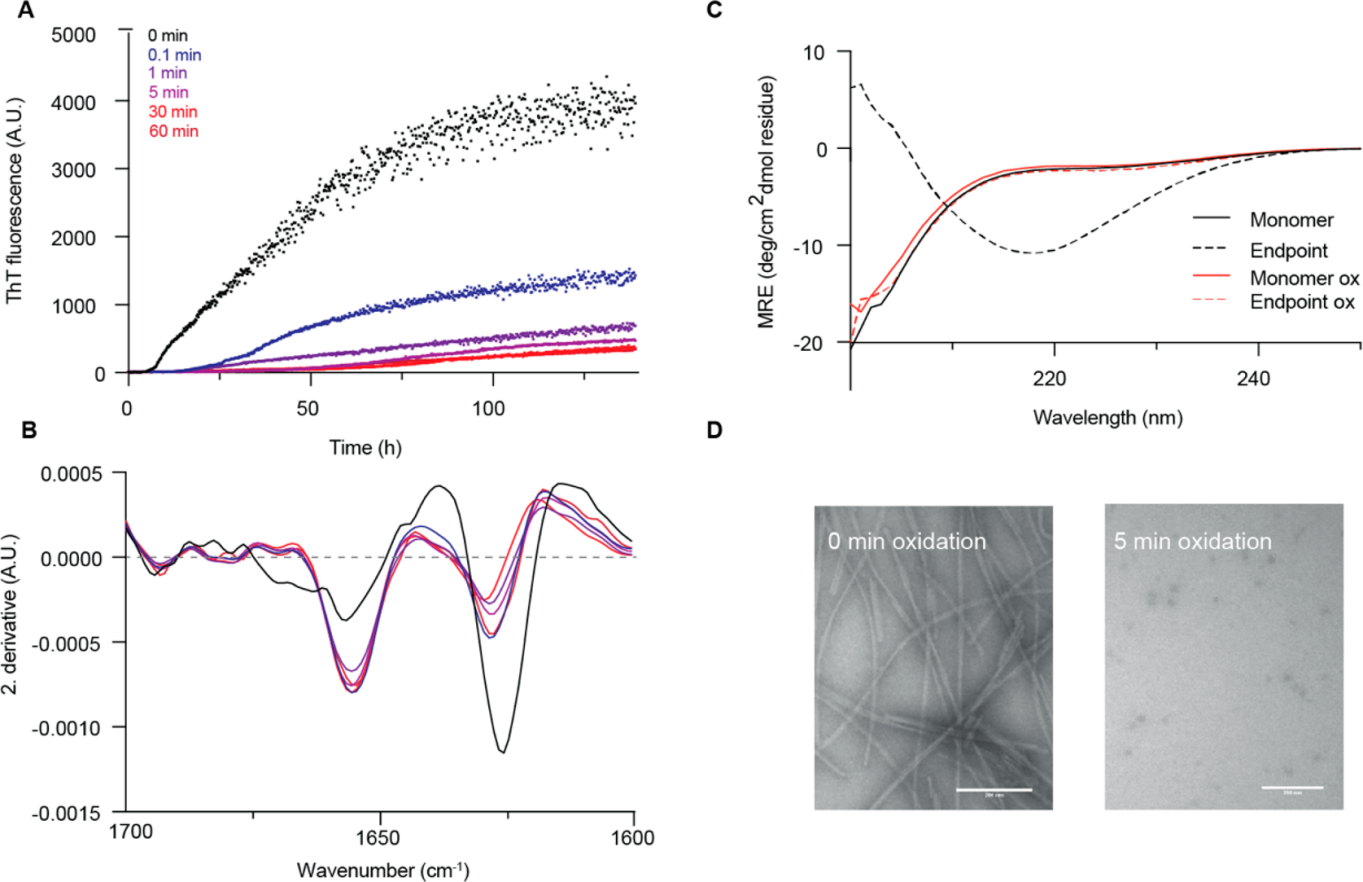
MCO inhibits amyloid formation. (A) ThT fluorescence was measured for α-Syn oxidized for 0, 0.1, 1, 5, 30, and 60 min prior to aggregation. (B) After the ThT signal had plateaued, the secondary structure was analyzed by FTIR, where the second derivative is shown for all time points. (C) CD spectra of untreated and 60 min oxidized monomer, and after reaching plateau in the ThT assay. (D) TEM images of untreated and oxidized α-Syn samples from the ThT assay. Scale bar: 200 nm.

In conclusion, we show that MCO associated with PD can induce long-range intramolecular diTyr cross-links which induce a compact, yet disordered, α-Syn monomer species. Steric hindrance from the diTyr linkage prevents aggregation of the monomer through β-sheet formation. Interference with the extended conformation of α-Syn opens up new interpretations of α-Syn function and pathology, as well as strategies to prevent α-Syn aggregation and ultimately treat PD.

## Supporting Information Available

Additional discussion, experimental data, and materials and methods (PDF)

## Abbreviations

α-Syn: α-synuclein
ATD: arrival time distribution
CIU: collision-induced unfolding
CCS: collision cross section
CD: circular dichroism
FTIR: Fourier transformed infrared spectroscopy
IM-MS: ion mobility mass spectrometry
MS: mass spectrometry
MCO: metal-catalyzed oxidation
PD: Parkinson’s disease
*R*_g_: radius of gyration
SAXS: small-angle X-ray scattering
ThT: thioflavin T
