# Supplementary material for: Structural Basis for Dityrosine-Mediated Inhibition of α-Synuclein Fibrillization
Source: J Am Chem Soc. 2022 Jun 24;144(27):11949–54. doi: 10.1021/jacs.2c03607 (PMC9284551; doi:10.1021/jacs.2c03607)
Supplement: Supplementary file 1 — ja2c03607_si_001.pdf [file ja2c03607_si_001.pdf]

Structural basis for dityrosine-mediated inhibition of α-synuclein fibrillization

Cagla Sahin*[a,b,1], Eva Christina Østerlund[c], Nicklas Österlund[d], Joana Costeira-Paulo[e,2], Jannik Nedergaard Pedersen[a], Gunna Christiansen[f], Janni Nielsen[a], Anne Louise Grønnemose[a,c,3], Søren Kirk Amstrup[a,b], Manish K. Tiwari[g,4], R. Shyama Prasad Rao[h,5], Morten Jannik Bjerrum[g], Leopold L. Ilag[i], Michael J. Davies[j], Erik G. Marklund[e], Jan Skov Pedersen[a,k], Michael Landreh[l], Ian Max Møller[m], Thomas J. D. Jørgensen*[c], Daniel Erik Otzen*[a,b]

a, Interdisciplinary Nanoscience Center (iNANO), Aarhus University, Gustav Wieds Vej 14, DK-8000 Aarhus C, Denmark

b, Department of Molecular Biology and Genetics, Aarhus University, Universitetsbyen 81, DK-8000 Aarhus C, Denmark

c, Department of Biochemistry and Molecular Biology, University of Southern Denmark, Campusvej 55, DK-5230 Odense M, Denmark

d, Department of Biochemistry and Biophysics, Stockholm University, SE-114 18 Stockholm, Sweden

e, Department of Chemistry – BMC, Uppsala University, Box 576, SE-751 23 Uppsala, Sweden

f, Department of Health Science and Technology, Medical Microbiology and Immunology, Aalborg University, Fredrik Bajers Vej 3b, DK-9220 Aalborg Ø, Denmark

g, Department Chemistry, University of Copenhagen, Universitetsparken 5, DK-2100 Copenhagen Ø, Denmark

h, Biostatistics and Bioinformatics Division, Yenepoya Research Center, Yenepoya University, Mangaluru 575018, Karnataka, India

i, Department of Materials and Environmental Chemistry, Stockholm University, SE-114 18 Stockholm, Sweden

j, Department of Biomedical Sciences, University of Copenhagen, Blegdamsvej 3B, DK-2200 Copenhagen N, Denmark

k, Department of Chemistry, Aarhus University, Gustav Wieds Vej 14, DK-8000 Aarhus C, Denmark

l, Department of Microbiology, Tumor and Cell Biology, Karolinska Institutet, Solnavägen 9, SE-171 65 Solna, Sweden

m, Department of Molecular Biology and Genetics, Aarhus University, Forsøgsvej 1, DK-4200 Slagelse, Denmark

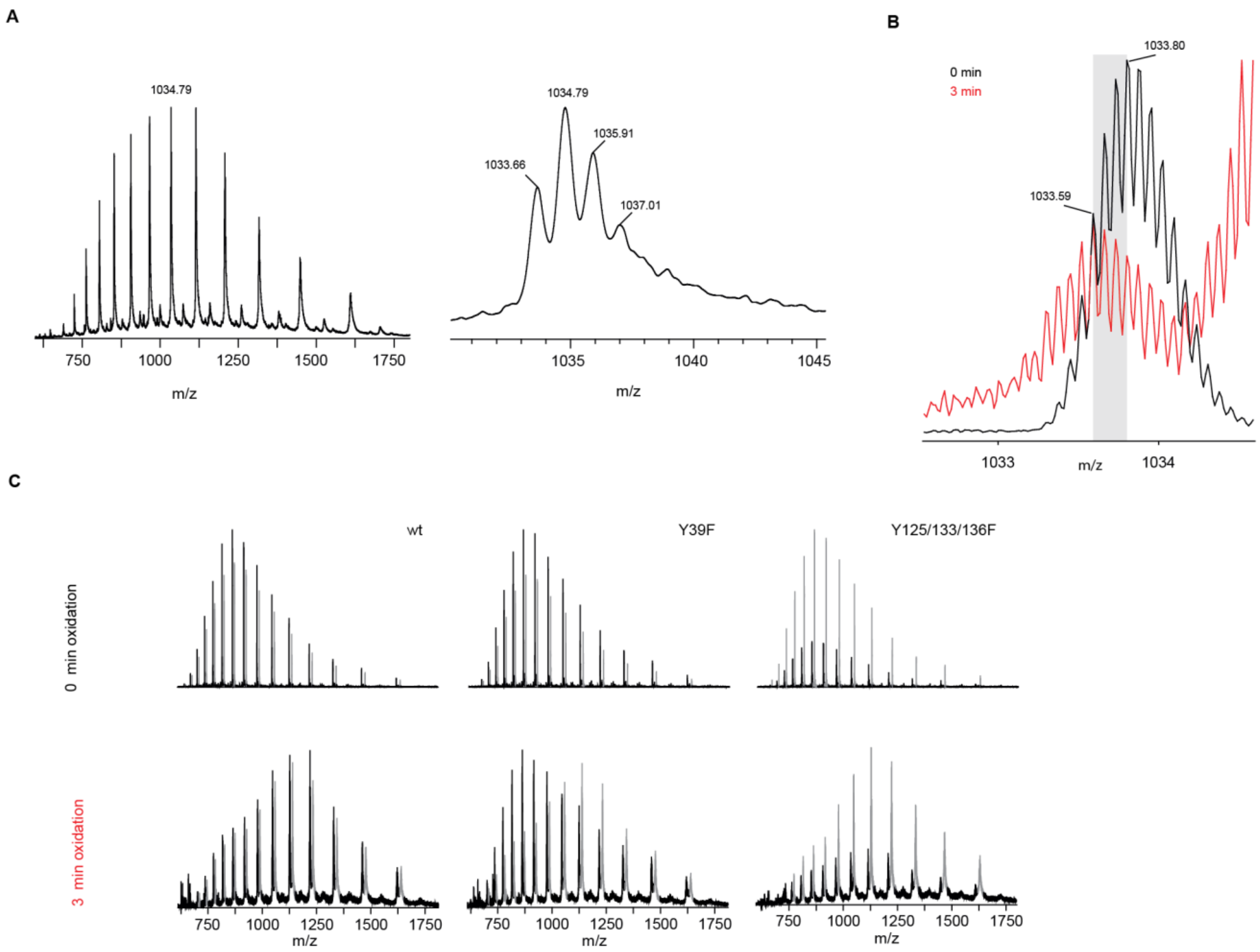

Figure S1. MS spectra showing oxidative modifications of α-Syn. A) Oxidation of wt α-Syn. Charge state distribution is seen to the left, and a zoom of the 14+ charge state showing +16 Da adducts. B) Overlay of charge state +14 from wt α-Syn (black) and wt α-Syn quenched after 3 min oxidation (red), where a reduction of 0.21 Da/charge is observed corresponding to a loss of 2.94 Da. C) ESI-MS charge state distribution of α-Syn wt, Y39F, Y125/133/136F spiked with ^{15}N-α-Syn (gray) as an internal control.

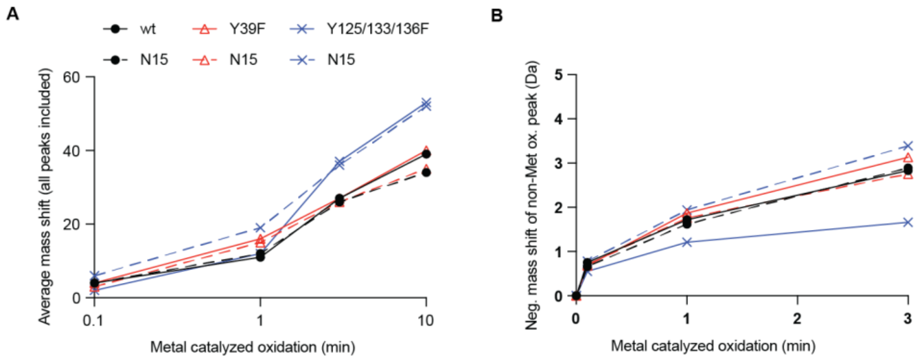

Figure S2. Mass shifts as a function of MCO oxidation of α-Syn mutants. ^{15}N-α-Syn was used as internal control to be able to compare the level of oxidation at short time points. A) Rate of methionine oxidation are not affected by mutating tyrosines to phenylalanine, B) whereas the triple mutant exhibits the lowest negative mass shift, yet still a negative mass shift. The loss of mass for the triple mutant is most likely due to crosslinks other than diTyr. Removing three potential crosslinking sites in the C-terminal, reduces the likelihood of forming intra-molecular crosslinks.

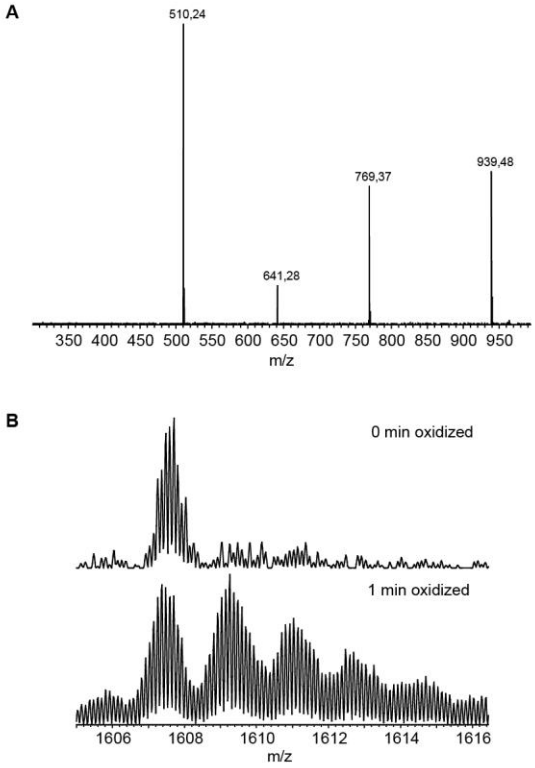

Figure S3. Using tandem MS, we eliminated the possibility of a loss of 2 Da upon formation of dehydromethionine of Met1. MS/MS analysis of oxidized alpha-synuclein reveals that the N-terminal methionine is not converted to dehydromethionine. (A) ETD spectrum of the +9 charge state of the alpha-synuclein oxidation product with mass shift of -2 Da that was generated by metal-catalyzed oxidation for 1 min. The masses of the N-terminal fragment ions are identical to the theoretical masses of the c-ions from non-oxidized alpha-synuclein thereby demonstrating that the N-terminal residues MDVFMKGL are not modified. (B) upper panel: MS spectrum displaying the isotope distribution of the +9 charge state of non-oxidized alpha-synuclein; lower panel: oxidized alpha-synuclein, the + 9 charge state at m/z 1607.37 was selected for ETD.

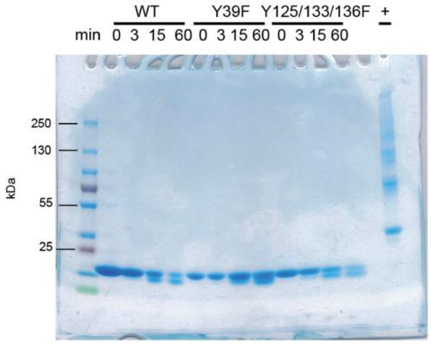

Figure S4. The whole SDS-PAGE from fig. 1D stained with Coomassie brilliant blue.

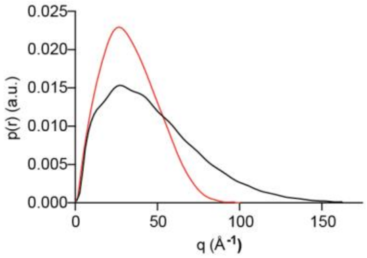

Figure S5. SAXS analysis of wt α-Syn and oxidized α-Syn. A) Pair distance distributions of untreated (black) and 60 min oxidized (red) α-Syn. The compaction one will observe between an ideal chain and ideal ring, according to statistical physics[1], which is based on calculation of radius of gyration depending on Kuhn length and number of segments. The Rg of an ideal chain is described by sqrt(N)*l/sqrt (6), whereas an ideal ring is described by sqrt(N)*l/sqrt(12).

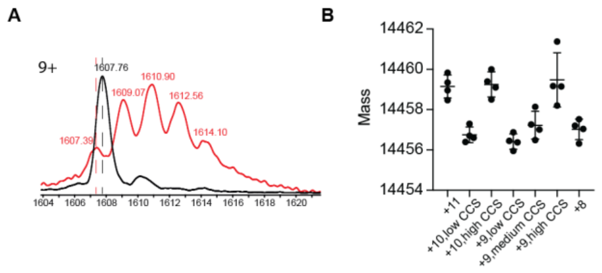

Figure S6 Ion mobility drift time-resolved masses of alpha-synuclein species with different CCS shown in Fig. 3B. (A) Drift time resolved MS spectra of oxidized wt α-Syn. Overlay of 5-min oxidized α-Syn (red) and native α-Syn (black) drift times of the +9 charge state. The positions of the unmodified α-Syn peaks are marked with a dashed line to highlight the mass shift. (B) The MS spectra were obtained after 5 min metal-catalyzed oxidation of α-Syn and they displayed a distribution of peaks corresponding to α-Syn with 1-4 methionine sulfoxides (mass of non-methionine oxidized α-Syn + n×16.0 Da, n = 1-4). To remove the mass increase that results from methionine oxidation, the masses for each CCS species were accordingly adjusted by subtraction of n×16.0 Da, n = 1-4. In this way, transformed masses of drift time resolved populations were obtained. Error bars are shown as ± one standard deviation. The masses corresponding to +11, +10 (high CCS), +9 (high CCS) are in good agreement with the theoretical mass of non-oxidized αSN (14460.0 Da) within measurement uncertainty. The masses corresponding to low or medium CCS species and +8 are all significantly lower than the high CCS masses and +11. This correlation between CCS and mass corroborates that the negative mass shift is due to the formation of dityrosine cross-link which in turn yields a more compact conformation with a lower CCS.

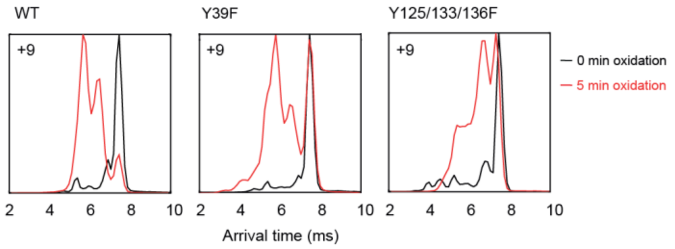

Figure S7. IM-MS analysis of mutant α-Syn. Arrival time distribution of the +9 charge states of untreated (black) and 5-min oxidized (red) wt, Y39F and Y125/133/136F α-Syn under denaturing conditions (50 % acetonitrile, 0.1 % formic acid).

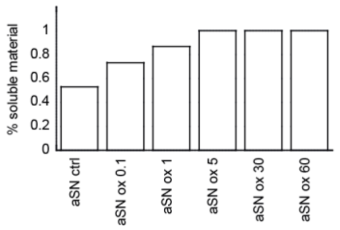

Figure S8. Soluble α-Syn after fibrillation. The amount of soluble α-Syn oxidized to 0, 0.1, 1, 3, 5, 15, 30 and 60 min after fibrillation (as per ThT assay), analyzed by SDS-PAGE and quantified by ImageJ. Normalized to non-fibrillated α-Syn (starting material).

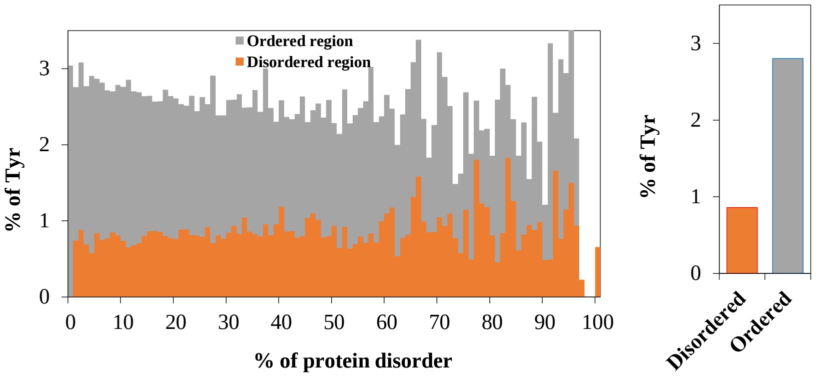

Figure S9. Bioinformatic analysis of Tyr residues in α-Syn. Tyr has a lower occurrence in IDPs than in other proteins indicating an important role for Tyr, *e.g.* to stabilize a crosslinked conformation. (A) Plot shows the percentage of Tyr residues in disordered and ordered regions versus the percentage of protein disorder. On average, disordered regions have far less Tyr (~0.86%) compared to ordered regions (~2.8%). Proportion of Tyr also slightly decreases in proteins as % disorder increases. Noisy pattern at higher % disorder is due to small sample size.

```
tr|A0A2H4ZEZ2_SOMMI        MDVFMKGLSKAKDGVVAAAEKTKQGVAEAAGKTKDGVLYMGSKTKEGVFHGVSSVAEKTK        60
tr|K9IGD3_DESRO            MDVLMKGLSKAKEGVVAAAEKTKQGVAEAAGKTKEGVLYVGSKTKEGVVHGVTTVAEKTK        60
tr|A0A2K6FYB9_PROCO        MDIFKKGLSKAKEGVVAAAEKTKQGVAEAAGKTKEGVLYVGSKTKEGVVHGVTTVAEKTK        60
tr|G1U0V2_RABIT            MDVFMKGLSKAKEGVVAAAEKTKQGVAEAAGKTKEGVLYVGSKTKEGVVHGVTTVAEKTK        60
tr|A0A250Y9X3_CASCN        MDVFMKGLSKAKEGVVAAAEKTKQGVAEAAGKTKEGVLYVGSKTKEGVVHGVTTVAEKTK        60
sp|P61142|SYUA_MACFA       MDVFMKGLSKAKEGVVAAAEKTKQGVAEAAGKTKEGVLYVGSKTKEGVVHGVATVAEKTK        60
sp|P61143|SYUA_MACMU       MDVFMKGLSKAKEGVVAAAEKTKQGVAEAAGKTKEGVLYVGSKTKEGVVHGVATVAEKTK        60
tr|A0A2K6APH5_MACNE        MDVFMKGLSKAKEGVVAAAEKTKQGVAEAAGKTKEGVLYVGSKTKEGVVHGVATVAEKTK        60
sp|P61139|SYUA_ERYPA       MDVFMKGLSKAKEGVVAAAEKTKQGVAEAAGKTKEGVLYVGSKTKEGVVHGVATVAEKTK        60
tr|A0A2K5KEA9_COLAP        MDVFMKGLSKAKEGVVAAAEKTKQGVAEAAGKTKEGVLYVGSKTKEGVVHGVATVAEKTK        60
tr|A0A2K6PRP9_RHIRO        MDVFMKGLSKAKEGVVAAAEKTKQGVAEAAGKTKEGVLYVGSKTKEGVVHGVATVAEKTK        60
tr|A0A0D9QW44_CHLSB        MDVFMKGLSKAKEGVVAAAEKTKQGVAEAAGKTKEGVLYVGSKTKEGVVHGVATVAEKTK        60
tr|A0A2K6LK63_RHIBE        MDVFMKGLSKAKEGVVAAAEKTKQGVAEAAGKTKEGVLYVGSKTKEGVVHGVATVAEKTK        60
tr|L5KSB3_PTEAL            MDVFMKGLSKAKEGVVAAAEKTKQGVAEAAGKTKEGVLYVGSKTKEGVVHGVTTVAEKTK        60
tr|G1RW17_NOMLE            MDVFMKGLSKAKEGVVAAAEKTKQGVAEAAGKTKEGVLYVCSKTKEGVVHGVATVAEKTK        60
sp|P61146|SYUA_PONAB       MDVFMKGLSKAKEGVVAAAEKTKQGVAEAAGKTKEGVLYVGSKTKEGVVHGVATVAEKTK        60
sp|P61140|SYUA_GORGO       MDVFMKGLSKAKEGVVAAAEKTKQGVAEAAGKTKEGVLYVGSKTKEGVVHGVATVAEKTK        60
**sp|P37840|SYUA_HUMAN     MDVFMKGLSKAKEGVVAAAEKTKQGVAEAAGKTKEGVLYVGSKTKEGVVHGVATVAEKTK**      60
sp|P61144|SYUA_PANPA       MDVFMKGLSKAKEGVVAAAEKTKQGVAEAAGKTKEGVLYVGSKTKEGVVHGVATVAEKTK        60
sp|P61145|SYUA_PANTR       MDVFMKGLSKAKEGVVAAAEKTKQGVAEAAGKTKEGVLYVGSKTKEGVVHGVATVAEKTK        60
sp|Q3T0G8|SYUA_BOVIN       MDVFMKGLSKAKEGVVAAAEKTKQGVAEAAGRTKEGVLYVGSKTKEGVVHGVTTVAEKTK        60
tr|L8I3Y5_9CETA            MDVFMKGLSKAKEGVVAAAEKTKQGVAEAAGRTKEGVLYVGSKTKEGVVHGVTTVAEKTK        60
tr|G3T7Z3_LOXAF            MDVFMKGLSKAKEGVVAAAEKTKQGVAEAAGKTKEGVLYVGSKTKEGVVHGVTTVAEKTK        60
tr|A0A2I2V101_FELCA        MDVFMKGLSKAKEGVVAAAEKTKQGVAEAAGKTKEGVLYVGSKTKEGVVHGVTTVAEKTK        60
tr|U6DYL0_NEOVI            --------------VVAAAEKTKQGVAEAAGKTKEGVLYVGSKTKEGVVHGVTTVAEKTK        46
tr|F6U044_HORSE            MDVFMKGLSKAKEGVVAAAEKTKQGVAEAAGKTKEGVLYVGSKTKEGVVHGVTTVAEKTK        60
tr|J7H0X3_TUPCH            MDVFMKGLSKAKEGVVAAAEKNKQGVAEAAGKTKEGVLYVGSKTKEGVVHGVTTVAEKTK        60
```

```
tr|A0A1L4APK6_BALMY     MDVFMKGLSKAKEGVVAAAEKTKQGVAEAAEKTKEGVLYVGSKTKEGVVHGVTTVAEKTK    60
tr|G1EFI2_SHEEP         MDVFMKGLSKAKEGVVAAAEKTKQGVAEAAGKTKEGVLYVGSKTKEGVVHGVTTVAEKTK    60
tr|M3Z596_MUSPF         MDVFMKGLSKAKEGVVAAAEKTKQGVAEAAGKTKEGVLYVGSKTKEGVVHGVTTVAEKTK    60
tr|E2RDD9_CANLF         MDVFMKGLSKAKEGVVAAAEKTKQGVAEAAGKTKEGVLYVGSKTKEGVVHGVTTVAEKTK    60
tr|G1M951_AILME         MDVFMKGLSKAKEGVVAAAEKTKQGVAEAAGKTKEGVLYVGSKTKEGVVHGVTTVAEKTK    60
tr|A0A2H4ZEZ3_URSMA     MDVFMKGLSKAKEGVVAAAEKTKQGVAEAAGKTKEGVLYVGSKTKEGVVHGVTTVAEKTK    60
sp|Q3I5G7|SYUA_PIG      MDVFMKGLSKAKEGVVAAAEKTKQGVAEAAGKTKEGVLYVGSKTKEGVVHGVTTVAEKTK    60
tr|A0A1L2YZJ0_GLOME     MDVFMKGLSKAKEGVVAAAEKTKQGVAEAAEKTKEGVLYVGSKTKEGVVHGVTTVAEKTK    60
tr|A0A1V4JTX8_PATFA     ----MKGLSKAKEGVVAAAEKTKQGVAEAAGKTKEGVLYVGSRTKEGVVHGVTTVAEKTK    56
tr|A4IH15_XENTR         MDVFMKGLSKAKEGVVAAAEKTKQGVAEAAGKTKEGVLYVGSKTKEGVVHGVTTVAEKTK    60
tr|Q7SZ02_XENLA         MDVFMKGLSKAKEGVVAAAEKTKQGVAEAAGKTKEGVLYVGSKTKEGVVHGVTTVAEKTK    60
tr|K7FW98_PELSI         MDVFMKGLSKAKEGVVAAAEKTKQGMAEAAGKTKEGVLYVGSRTREGVVHGVTTMAEKTK    60
tr|H0VPZ2_CAVPO         MDVFMKGLSKAKEGVVAAAEKTKQGVAEAAGKTKEGVLYVGSKTKEGVVHGVTTVAEKTK    60
tr|A0A2K5LDP9_CERAT     MDVFMKGLSKAKEGVVAAAEKTKQGVAEAAGKTKEGVLYVGSKTKEGVVHGVATVAEKTK    60
tr|A0A2I3N0Z9_PAPAN     MDVFMKGLSKAKEGVVAAAEKTKQGVAEAAGKTKEGVLYVGSKTKEGVVHGVATVAEKTK    60
tr|A0A2K5XH75_MANLE     MDVFMKGLSKAKEGVVAAAEKTKQGVAEAAGKTKEGVLYVGSKTKEGVVHGVATVAEKTK    60
tr|A0A1S3GAZ7_DIPOR     MDVFMKGLSKAKEGVVAAAEKTKQGVAEAAGKTKEGVLYVGSKTKEGVVHGVTTVAEKTK    60
tr|A0A2K5PEF0_CEBCA     MDVFMKGLSKAKEGVVAAAEKTKQGVAEAAGKTKEGVLYVGSKTKEGVVHGVATVAEKTK    60
tr|I3MD35_ICTTR         MDVFMKGLSKAKEGVVAAAEKTKQGVAEAAGKTKEGVLYVGSKTKEGVVHGVTTVAEKTK    60
sp|P61138|SYUA_ATEGE    MDVFMKGLSKAKEGVVAAAEKTKQGVAEAAGKTKEGVLYVGSKTKEGVVHGVTTVAEKTK    60
sp|P61141|SYUA_LAGLA    MDVFMKGLSKAKEGVVAAAEKTKQGVAEAAGKTKEGVLYVGSKTKEGVVHGVTTVAEKTK    60
sp|P61147|SYUA_SAGLB    MDVFMKGLSKAKEGVVAAAEKTKQGVAEAAGKTKEGVLYVGSKTKEGVVHGVTTVAEKTK    60
tr|F7GY62_CALJA         MDVFMKGLSKAKEGVVAAAEKTKQGVAEAAGKTKEGVLYVGSKTKEGVVHGVTTVAEKTK    60
tr|A0A2K5CEE9_AOTNA     MDVFMKGLSKAKEGVVAAAEKTKQGVAEAAGKTKEGVLYVGSKTKEGVVHGVTTVAEKTK    60
tr|A0A0A7HRT6_HETGA     MDVFMKGLSKAKEGVVAAAEKTKQGVAEAAGKTKEGVLYVGSKTKEGVVHGVTTVAEKTK    60
tr|A0A2K6SI59_SAIBB     MDVFMKGLSKAKEGVVAAAEKTKQGVAEAAGKTKEGVLYVGSKTKEGVVHGVATVAEKTK    60
tr|D0FH84_SAISC         MDVFMKGLSKAKEGVVAAAEKTKQGVAEAAGKTKEGVLYVGSKTKEGVVHGVATVAEKTK    60
sp|P37377|SYUA_RAT      MDVFMKGLSKAKEGVVAAAEKTKQGVAEAAGKTKEGVLYVGSKTKEGVVHGVTTVAEKTK    60
sp|O55042|SYUA_MOUSE    MDVFMKGLSKAKEGVVAAAEKTKQGVAEAAGKTKEGVLYVGSKTKEGVVHGVTTVAEKTK    60
tr|A0A1U8C123_MESAU     MDVFMKGLSKAKEGVVAAAEKTKQGVAEAAGKTKEGVLYVGSKTKEGVVHGVTTVAEKTK    60
tr|A0A218V256_9PASE     MDVFMKGLSKAKEGVVAAAEKTKQGVAEAAGKTKEGVLYVGSRTKEGVVHGVTTVAEKTK    60
sp|Q91448|SYUA_SERCA    MDVFMKGLSKAKEGVVAAAEKTKQGVAEAAGKTKEGVLYVGSRTKEGVVHGVTTVAEKTK    60
tr|A0A1U7RPT7_ALLSI     MDVFMKGLSKAKEGVVAAAEKTKQGVAEAAGKTKEGVLYMGSRTKEGVVHGVTTVAEKTK    60
tr|A0A099ZN27_TINGU     MDVFMKGLSKAKEGVVAAAEKTKQGVAEAAGKTKEGVLYVGSRTKEGVVHGVTTVAEKTK    60
tr|Q4JHT6_TAEGU         MDVFMKGLSKAKEGVVAAAEKTKQGVAEAAGKTKEGVLYVGSRTKEGVVHGVTTVAEKTK    60
tr|U3JU64_FICAL         MDVFMKGLSKAKEGVVAAAEKTKQGVAEAAGKTKEGVLYVGSRTKEGVVHGVTTVAEKTK    60
tr|A0A2I0M939_COLLI     MDVFMKGLSKAKEGVVAAAEKTKQGVAEAAGKTKEGVLYVGSRTKEGVVHGVTTVAEKTK    60
tr|A0A091FLC8_9AVES     MDVFMKGLSKAKEGVVAAAEKTKQGVAEAAGKTKEGVLYVGSRTKEGVVHGVTTVAEKTK    60
tr|A0A093FVV3_DRYPU     MDVFMKGLSKAKEGVVAAAEKTKQGVAEAAGKTKEGVLYVGSRTKEGVVHGVTTVAEKTK    60
tr|A0A087QQY8_APTFO     MDVFMKGLSKAKEGVVAAAEKTKQGVAEAAGKTKEGVLYVGSRTKEGVVQGVTTVAEKTK    60
tr|R4GAF5_ANOCA         MDVFMKGFNKAKDGVVAAAEKTKQGVAEAAGKTKEGVLYVGSKTRDGVVQGVTTVAEKTK    60
tr|A0A091I049_CALAN     MDVFMKGLSKAKEGVVAAAEKTKQGVAEAAGKTKEGVLYVGSRTKEGVVHGVTTVAEKTK    60
```

```
tr|A0A091F433_CORBR      MDVFMKGLSKAKEGVVAAAEKTKQGVAEAAGKTKEGVLYVGSRTKEGVVHGVTTVAEKTK      60
tr|A0A093PG66_PYGAD      MDVFMKGLSKAKEGVVAAAEKTKQGVAEAAGKTKEGVLYVGSRTKEGVVHGVTTVAEKTK      60
tr|A0A091JLR4_EGRGA      MDVFMKGLSKAKEGVVAAAEKTKQGVAEAAGKTKEGVLYVGSRTKEGVVHGVTTVAEKTK      60
tr|A0A091WCU5_OPIHO      MDVFMKGLSKAKEGVVAAAEKTKQGVAEAAGKTKEGVLYVGSRTKEGVVHGVTTVAEKTK      60
tr|A0A091X556_NIPNI      MDVFMKGLSKAKEGVVAAAEKTKQGVAEAAGKTKEGVLYVGSRTKEGVVHGVTTVAEKTK      60
tr|A0A093QA93_9PASS      MDVFMKGLSKAKEGVVAAAEKTKQGVAEAAGKTKEGVLYVGSRTKEGVVHGVTTVAEKTK      60
tr|A0A0A0ANP9_CHAVO      MDVFMKGLSKAKEGVVAAAEKTKQGVAEAAGKTKEGVLYVGSRTKEGVVHGVTTVAEKTK      60
tr|A0A093JQV1_STRCA      MDVFMKGLSKAKEGVVAAAEKTKQGVAEAAGKTKEGVLYVGSRTKEGVVHGVTTVAEKTK      60
tr|A0A093CC60_TAUER      MDVFMKGLSKAKEGVVAAAEKTKQGVAEAAGKTKEGVLYVGSRTKEGVVHGVTTVAEKTK      60
tr|Q9I9H1_CHICK          MDVFMKGLNKAKEGVVAAAEKTKQGVAEAAGKTKEGVLYVGSRTKEGVVHGVTTVAEKTK      60
tr|G1N1K5_MELGA          MDVFMKGLNKAKEGVVAAAEKTKQGVAEAAGKTKEGVLYVGSRTKEGVVHGVTTVAEKTK      60
tr|A0A2D4GM30_MICCO      MDVFMKGFNKAKEGMVAAAEKTKQGVAEAAGKTKEGVLYVGSKTKEGVVHGVTTVAEKTK      60
                                       :******.***:**** :**:****: *:*::**.:**:::*****

tr|A0A2H4ZEZ2_SOMMI      EQASLVGGAVVTGMSTVAQKTVEGAGNIAAATGLVKKEQLVKQDEDLFPQEGAMDNTEAL      120
tr|K9IGD3_DESRO          EQVTNVGEAVVTGVTAVAQKTVEGAGNIAAATGFGRKDQLGKSE-EGAPQEGIL---EDM      116
tr|A0A2K6FYB9_PROCO      EQVTNVGGAVVTGVTAVAQKTVEGAGSIAAATGFGKKDQFGKSE-EGAPQEGIL---EDT      116
tr|G1U0V2_RABIT          EQVTNVGEAVVTGVTAVAQKTVEGAGSIAAATGFVKKDQQGKSE-EGAPQEGIL---EAM      116
tr|A0A250Y9X3_CASCN      EQVTNVGGAVVTGVTAVAHKTVEGAGSIAAATGFVKKDQLGKSE-EGSPQEGIL---EDM      116
sp|P61142|SYUA_MACFA     EQVTNVGGAVVTGVTAVAQKTVEGAGSIAAATGFIKKDQLGKNE-EGAPQEGIL---QDM      116
sp|P61143|SYUA_MACMU     EQVTNVGGAVVTGVTAVAQKTVEGAGSIAAATGFIKKDQLGKNE-EGAPQEGIL---QDM      116
tr|A0A2K6APH5_MACNE      EQVTNVGGAVVTGVTAVAQKTVEGAGSIAAATGFIKKDQLGKNE-EGAPQEGIL---QDM      116
sp|P61139|SYUA_ERYPA     EQVTNVGGAVVTGVTAVAQKTVEGAGSIAAATGFVKKDQLGKNE-EGAPQEGIL---QDM      116
tr|A0A2K5KEA9_COLAP      EQVTNVGGAVVTGVTAVAQKTVEGAGSIAAATGFVKKDQLGKNE-EGAPQEGIL---QDM      116
tr|A0A2K6PRP9_RHIRO      EQVTNVGGAVVTGVTAVAQKTVEGAGSIAAATGFVKKDQLGKNE-EGAPQEGIL---QDM      116
tr|A0A0D9QW44_CHLSB      EQVTNVGGAVVTGVTAVAQKTVEGAGSIAAATGFVKKDQLGKNE-EGAPQEGIL---QDM      116
tr|A0A2K6LK63_RHIBE      EQVTNVGGAVVTGVTAVAQKTVEGAGSIAAATGFVKKDQLGKNE-EGAPQEGIL---QDM      116
tr|L5KSB3_PTEAL          EQVTNVGEAVVTGVTAVAQKTVEGAGSIAAATGFGRKDQLGKSE-EGAPQEGIL---EDM      116
tr|G1RW17_NOMLE          EQVTNVGGAVVTGVTAVAQKTVEGAGSIAAATGFVKKDQLGKSE-EGAPQEGIL---EDM      116
sp|P61146|SYUA_PONAB     EQVTNVGGAVVTGVTAVAQKTVEGAGSIAAATGFVKKDQLGKNE-EGATQEGIL---EDM      116
sp|P61140|SYUA_GORGO     EQVTNVGGAVVTGVTAVAQKTVEGAGSIAAATGFVKKDQLGKNE-EGAPQEGIL---EDM      116
sp|P37840|SYUA_HUMAN     EQVTNVGGAVVTGVTAVAQKTVEGAGSIAAATGFVKKDQLGKNE-EGAPQEGIL---EDM      116
sp|P61144|SYUA_PANPA     EQVTNVGGAVVTGVTAVAQKTVEGAGSIAAATGFVKKDQLGKNE-EGAPQEGIL---EDM      116
sp|P61145|SYUA_PANTR     EQVTNVGGAVVTGVTAVAQKTVEGAGSIAAATGFVKKDQLGKNE-EGAPQEGIL---EDM      116
sp|Q3T0G8|SYUA_BOVIN     EQVTNVGEAVVTGVTAVAQKTVEGAGSIAAATGFGKKDHMGKGE-EGASQEGIL---EDM      116
tr|L8I3Y5_9CETA          EQVTNVGEAVVTGVTAVAQKTVEGAGSIAAATGFGKKDHMGKGE-EGASQEGIL---EDM      116
tr|G3T7Z3_LOXAF          EQVTNVGEAVVTGVTAVAQKTVEGAGSIAAATGFGKKDQMGKGE-EGAPQEGIL---ENV      116
tr|A0A2I2V101_FELCA      EQVTNVGEAVVTGVTAVAQKTVEGAGSIAAATGFGKKDQLGKNE-EGGPQEGIL---EDM      116
tr|U6DYL0_NEOVI          EQVTNVGEAVVTGVTAVAQKTVEGAGSIAAATGFGKKDQLGKSE-EGGPQEGIL---EDM      102
tr|F6U044_HORSE          EQVTNVGEAVVTGVTAVAQKTVEGAESIAAATGFGKKDHLGKSE-EGAAQEGIL---EDM      116
tr|J7H0X3_TUPCH          EQVTNVGGAVVTGVTAVAQKTVEGAGSIAAATGFGKKDQLGKSE-EGAPQEGIL---EDM      116
tr|A0A1L4APK6_BALMY      EQVTNVGEAVVTGVTAVAQKTVEGAGSIAAATGFGKKDQLGKS--EGASQEGIL---EDT      115
```

tr|G1EFI2_SHEEP	EQVTNVGEAVVTGVTAVAQKTVEGAGSIAAATGFGKKDHLGKGE-EGASQEGIL---EDM	116
tr|M3Z596_MUSPF	EQVTNVGEAVVTGVTAVAQKTVEGAGSIAAATGFGKKDQLGKSE-EGGPQEGIL---EDM	116
tr|E2RDD9_CANLF	EQVTNVGEAVVTGVTAVAQKTVEGAGSIAAATGFGKKDQLGKSE-EGGPQEGIL---EDM	116
tr|G1M951_AILME	EQVTNVGEAVVTGVTAVAQKTVEGAGSIAAATGFGKKDQLGKSE-EGGPQEGIL---EDM	116
tr|A0A2H4ZEZ3_URSMA	EQVTNVGEAVVTGVTAVAQKTVEGAGSIAAATGFGKKDQLGKSE-EGGPQEGIL---EDM	116
sp|Q3I5G7|SYUA_PIG	EQVTNVGEAVVTGVTAVAQKTVEGAGSIAAATGFGKKDQLGKNE-EGAPQEGIL---EDM	116
tr|A0A1L2YZJ0_GLOME	EQVTNVGEAVVTGVTAVAQKTVEGAGSIAAATGFGKKDQLGKS--EGASQEGIL---EDT	115
tr|A0A1V4JTX8_PATFA	EQVSNVGGAVVTGVTAVAQKTVEGAGNIAAATGLVKKDPLAKQNEEGFLQEGMV-NNADV	115
tr|A4IH15_XENTR	EQVSNVGGAVVTGVTAVAQKTVEGAGNIAAATGLVKKDQK---NESGFGQEGTVENSENM	117
tr|Q7SZ02_XENLA	EQVSNVGGAVVTGVTAVAHKTVEGAGNFAAATGLVKKDQK---NESGFGPEGTMENSENM	117
tr|K7FW98_PELSI	EQVSNVGGAVVTGVTAVAHKTVEGAGNIAAATGLVKKDQMAKQNEEGLSQEGMM-DSTDM	119
tr|H0VPZ2_CAVPO	EQVTNVGGAVVTGVTAVAHKTVEGAGNIAAATGFVRKDQLGKNE-EGSSQEGIL---EDM	116
tr|A0A2K5LDP9_CERAT	EQVTNVGGAVVTGVTAVAQKTVEGAGSIAAATGFVKKDQLGKNE-EGTPQEGIL---QDM	116
tr|A0A2I3N0Z9_PAPAN	EQVTNVGGAVVTGVTAVAQKTVEGAGSIAAATGFVKKDQLGKNE-EGTPQEGIL---QDM	116
tr|A0A2K5XH75_MANLE	EQVTNVGGAVVTGVTAVAQKTVEGAGSIAAATGFVKKDQLGKNE-EGTPQEGIL---QDM	116
tr|A0A1S3GAZ7_DIPOR	EQVTNVGGAVVTGVTTVAQKTVEGAGNIAAATGFVKKDQLGKSG-EGSPQEGIL---EDM	116
tr|A0A2K5PEF0_CEBCA	EQVTNVGGAVVTGVTAVAQKTVEGAGNIAAATGFVRKDHLSKSE-EGAPQEGIL---EDM	116
tr|I3MD35_ICTTR	EQVTNVGGAVVTGVTAVAQKTVEGAGNIAAATGFVKKDQLGKSE-EGSPQEGIL---EDM	116
sp|P61138|SYUA_ATEGE	EQVTSVGGAVVTGVTAVAQKTVEGAGNIAAATGFVKKDHSGKSE-EGAPQEGIL---EDM	116
sp|P61141|SYUA_LAGLA	EQVTSVGGAVVTGVTAVAQKTVEGAGNIAAATGFVKKDHSGKSE-EGAPQEGIL---EDM	116
sp|P61147|SYUA_SAGLB	EQVTNVGGAVVTGVTAVAQKTVEGAGNIAAATGFVRKDHLGKSE-EGAPQEGIL---EDM	116
tr|F7GY62_CALJA	EQVTNVGGAVVTGVTAVAQKTVEGAGNIAAATGFVKKDHLGKSE-EGAPQEGIL---EDM	116
tr|A0A2K5CEE9_AOTNA	EQVTNVGGAVVTGVTAVAQKTVEGAGNIAAATGFVKKDHLGKSE-EGAPQEGIL---EDM	116
tr|A0A0A7HRT6_HETGA	EQVTNVGGAVVTGVTAVAQKTVEGAGNIAAATGFVKKDQLGKSE-EGSPQEGIL---EDM	116
tr|A0A2K6SI59_SAIBB	EQVTNVGGAVVTGVTAVAQKTVEGAGNIAAATGFVKKDHLGKSE-EGAPQEGIL---EDM	116
tr|D0FH84_SAISC	EQVTNVGGAVVTGVTAVAQKTVEGAGNIAAATGFVKKDHLGKSE-EGAPQEGIL---EDM	116
sp|P37377|SYUA_RAT	EQVTNVGGAVVTGVTAVAQKTVEGAGNIAAATGFVKKDQMGKGE-EGYPQEGIL---EDM	116
sp|O55042|SYUA_MOUSE	EQVTNVGGAVVTGVTAVAQKTVEGAGNIAAATGFVKKDQMGKGE-EGYPQEGIL---EDM	116
tr|A0A1U8C123_MESAU	EQVTNVGGAVVTGVTAVAQKTVEGAGNIAAATGFVKKD---KGE-EGYPQEGIL---EDM	113
tr|A0A218V256_9PASE	EQVSNVGGAVVTGVTAVAQKTVEGAGNIAAATGLVKKDQLAKQNEEGFLQEGMV-NNTGV	119
sp|Q91448|SYUA_SERCA	EQVSNVGGAVVTGVTAVAQKTVEGAGNIAAATGLVKKDQLAKQNEEGFLQEGMV-NNTGA	119
tr|A0A1U7RPT7_ALLSI	EQVSNVGGAVVTGVTAVAQKTVEGAGNIAAATGLVKKDQLAKQNEEGIPQEGMM-DNTDM	119
tr|A0A099ZN27_TINGU	EQVSNVGGAVVTGVTAVAQKTVEGAGNIAAATGLVKKDQLAKQNEDGILQEGMV-NNTGV	119
tr|Q4JHT6_TAEGU	EQVSNVGGAVVTGVTAVAQKTVEGAGNIAAATGLVKKDQLAKQNEEGFLQEGMV-NNTGV	119
tr|U3JU64_FICAL	EQVSNVGGAVVTGVTAVAQKTVEGAGNIAAATGLVKKDQLAKQNEEGFLQEGMV-NNTGV	119
tr|A0A2I0M939_COLLI	EQVSNVGGAVVTGVTAVAQKTVEGAGNIAAATGLVKKDPLAKQNEEGFLQEGMV-NNADV	119
tr|A0A091FLC8_9AVES	EQVSNVGGAVVTGVTAVAQKTVEGAGNIAAATGLVKKDQLAKQNEEGFLQEGMV-NNTDV	119
tr|A0A093FVV3_DRYPU	EQVSNVGGAVVTGVTAVAQKTVEGAGNIAAATGLVKKDQLAKQNEEGFVQEGMV-NNADL	119
tr|A0A087QQY8_APTFO	EQVSNVGGAVVTGVTAVAQKTVEGAGNIAAATGLVKKDQLAKQNEEGFLQEGMV-NNTDV	119
tr|R4GAF5_ANOCA	EQVSNVGGAVVTGVTAVAQKTVEGAGNFAAATGFVKKDQLGKQD-EGLPQEGMM-ANTDM	118
tr|A0A091I049_CALAN	EQVSNVGGAVVTGVTAVAQKTVEGAGSIAAATGLVKKDQLGKQNEEGFLQEGMV-NNADV	119
tr|A0A091F433_CORBR	EQVSNVGGAVVTGVTAVAQKTVEGAGNIAAATGLVKKDQLAKQNEEGFLQEGMV-NNTDV	119

```
tr|A0A093PG66_PYGAD      EQVSNVGGAVVTGVTAVAQKTVEGAGNIAAATGLVKKDQLAKQNEEGFLQEGMV-NNTDV        119
tr|A0A091JLR4_EGRGA      EQVSNVGGAVVTGVTAVAQKTVEGAGNIAAATGLVKKDQLAKQNEEGFLQEGMV-NNTDV        119
tr|A0A091WCU5_OPIHO      EQVSNVGGAVVTGVTAVAQKTVEGAGNIAAATGLVKKDQLAKQNEEGFLQEGMV-NNTDV        119
tr|A0A091X556_NIPNI      EQVSNVGGAVVTGVTAVAQKTVEGAGNIAAATGLVKKDQLAKQNEEGFLQEGMV-NNTDV        119
tr|A0A093QA93_9PASS      EQVSNVGGAVVTGVTAVAQKTVEGAGNIAAATGLVKKDQLAKQNEEGFLQEGMV-NNSDV        119
tr|A0A0A0ANP9_CHAVO      EQVSNVGGAVVTGVTAVAQKTVEGAGNIAAATGLVKKDQLAKQNEEGFLQEGMM-NNTDV        119
tr|A0A093JQV1_STRCA      EQVSNVGGAVVTGVTAVAQKTVEGAGNIAAATGLVKKDQLAKQNEDGNPQEGLV-NNTGV        119
tr|A0A093CC60_TAUER      EQVSNVGGAVVTGVTAVAQKTVEGAGNIAAATGLVKKDQLGKQNEEGFLQEGMV-NNTDV        119
tr|Q9I9H1_CHICK          EQVSNVGGAVVTGVTAVAQKTVEGAGNIAAATGLVKKDQLAKQNEEGFLQEGMV-NNTDI        119
tr|G1N1K5_MELGA          EQVSNVGGAVVTGVTAVAQKTVEGAGNIAAATGLVKKDQLAKQNEEGFLQEGVV-NNTDI        119
tr|A0A2D4GM30_MICCO      EQVSNVGGAVVTGVTAVAQKTVEGAGNIAAATGFVKKDQLGKQNEQGLTPEGNT-----D        115
                         **.: ** *****:::**:****** .:*****: :*:        .    **


tr|A0A2H4ZEZ2_SOMMI      QIDPDSQAYGAAPEGEYQDYGPEA  144
tr|K9IGD3_DESRO          PGDPDNETYEMPSEEGYQDYEPEA  140
tr|A0A2K6FYB9_PROCO      PMDPDSEAYEMPSEEGYQDYEPEA  140
tr|G1U0V2_RABIT          PMDPDSEAYEMPSEEGYQDYEPEA  140
tr|A0A250Y9X3_CASCN      PMDPDNEAYEMPSEEGYQDYEPEA  140
sp|P61142|SYUA_MACFA     PVDPDNEAYEMPSEEGYQDYEPEA  140
sp|P61143|SYUA_MACMU     PVDPDNEAYEMPSEEGYQDYEPEA  140
tr|A0A2K6APH5_MACNE      PVDPDNEAYEMPSEEGYQDYEPEA  140
sp|P61139|SYUA_ERYPA     PVDPDNEAYEMPSEEGYQDYEPEA  140
tr|A0A2K5KEA9_COLAP      PVDPDNEAYEMPSEEGYQDYEPEA  140
tr|A0A2K6PRP9_RHIRO      PVDPDNEAYEMPSEEGYQDYEPEA  140
tr|A0A0D9QW44_CHLSB      PVDPDNEAYEMPSEEGYQDYEPEA  140
tr|A0A2K6LK63_RHIBE      PVDPDNEAYEMPSEEGYQDYEPEA  140
tr|L5KSB3_PTEAL          PMDPENETYEMPSEEGYQDYEPEA  140
tr|G1RW17_NOMLE          PVDPDNEAYEMPSEEGYQDYEPEA  140
sp|P61146|SYUA_PONAB     PVDPDNEAYEMPSEEGYQDYEPEA  140
sp|P61140|SYUA_GORGO     PVDPDNEAYEMPSEEGYQDYEPEA  140
sp|P37840|SYUA_HUMAN     PVDPDNEAYEMPSEEGYQDYEPEA  140
sp|P61144|SYUA_PANPA     PVDPDNEAYEMPSEEGYQDYEPEA  140
sp|P61145|SYUA_PANTR     PVDPDNEAYEMPSEEGYQDYEPEA  140
sp|Q3T0G8|SYUA_BOVIN     PVDPDNEAYEMPSEEGYQDYEPEA  140
tr|L8I3Y5_9CETA          PVDPDNEAYEMPSEEGYQDYEPEA  140
tr|G3T7Z3_LOXAF          PVDPDNEAYEMPSEEGYQDYEPEA  140
tr|A0A2I2V101_FELCA      PVDPDNEAYEMPSEEGYQDYEPEA  140
tr|U6DYL0_NEOVI          PVDPDNEAYEMPSEEGYQDYEPEA  126
tr|F6U044_HORSE          PVDPDNEAYEMPSEEGYQDYEPEA  140
tr|J7H0X3_TUPCH          PVDPDNEAYEMPSEEGYQDYEPEA  140
tr|A0A1L4APK6_BALMY      PVDPDNEAYEMPSEEGYQDYEPEA  139
tr|G1EFI2_SHEEP          PVDPDNEAYEMPSEEGYQDYEPEA  140
```

tr|M3Z596_MUSPF	PVDPDNEAYEMPSEEGYQDYEPEA	140
tr|E2RDD9_CANLF	PVDPDNEAYEMPSEEGYQDYEPEA	140
tr|G1M951_AILME	PVDPDNEAYEMPSEEGYQDYEPEA	140
tr|A0A2H4ZEZ3_URSMA	PVDPDNEAYEMPSEEGYQDYEPEA	140
sp|Q3I5G7|SYUA_PIG	PVDPDNEAYEMPSEEGYQDYEPEA	140
tr|A0A1L2YZJ0_GLOME	PVDPDNEAYEMPSEEGYQDYEPEA	139
tr|A0A1V4JTX8_PATFA	PVDPENEAYEMPPEEEYQDYEPEA	139
tr|A4IH15_XENTR	PVNPD-ETYEMPPEEEYQDYDPEA	140
tr|Q7SZ02_XENLA	PVNPNNETYEMPPEEEYQDYDPEA	141
tr|K7FW98_PELSI	PMDPDNEAYEMPPEEEYQDYEPEA	143
tr|H0VPZ2_CAVPO	PVDPDNEAYEMPSEEGYQDYEPEA	140
tr|A0A2K5LDP9_CERAT	PVDPDNEAYEMPSEEGYQDYEPEA	140
tr|A0A2I3N0Z9_PAPAN	PVDPDNEAYEMPSEEGYQDYEPEA	140
tr|A0A2K5XH75_MANLE	PVDPDNEAYEMPSEEGYQDYEPEA	140
tr|A0A1S3GAZ7_DIPOR	PVDPDNEAYEMPSEEGYQDYEPEA	140
tr|A0A2K5PEF0_CEBCA	PVDPDNEAYEMPSEEGYQDYEPEA	140
tr|I3MD35_ICTTR	PVEPDNEAYEMPSEEGYQDYEPEA	140
sp|P61138|SYUA_ATEGE	PVDPDNEAYEMPSEEGYQDYEPEA	140
sp|P61141|SYUA_LAGLA	PVDPDNEAYEMPSEEGYQDYEPEA	140
sp|P61147|SYUA_SAGLB	PVDPDNEAYEMPSEEGYQDYEPEA	140
tr|F7GY62_CALJA	PVDPDNEAYEMPSEEGYQDYEPEA	140
tr|A0A2K5CEE9_AOTNA	PVDPDNEAYEMPSEEGYQDYEPEA	140
tr|A0A0A7HRT6_HETGA	PVDPDNEAYEMPSEEGYQDYEPEA	140
tr|A0A2K6SI59_SAIBB	PVDPDNEAYEMPSEEGYQDYEPEA	140
tr|D0FH84_SAISC	PVDPDNEAYEMPSEEGYQDYEPEA	140
sp|P37377|SYUA_RAT	PVDPSSEAYEMPSEEGYQDYEPEA	140
sp|O55042|SYUA_MOUSE	PVDPGSEAYEMPSEEGYQDYEPEA	140
tr|A0A1U8C123_MESAU	PVEPGSEAYEMPSEEGYQDYEPEA	137
tr|A0A218V256_9PASE	AVGPENEAYKMPPEEEYQDYEPEA	143
sp|Q91448|SYUA_SERCA	AVDPDNEAYEMPPEEEYQDYEPEA	143
tr|A0A1U7RPT7_ALLSI	PVDTENEAYEMPPEEKYQDYEPEA	143
tr|A0A099ZN27_TINGU	AVDPENEAYEMPPEEEYQDYEPEA	143
tr|Q4JHT6_TAEGU	AVDPENEAYEMPPEEEYQDYEPEA	143
tr|U3JU64_FICAL	PVDPESEAYEMPPEEEYQDYEPEA	143
tr|A0A2I0M939_COLLI	PVDPENEAYEMPPEEEYQDYEPEA	143
tr|A0A091FLC8_9AVES	PVDPENEAYEMPPEEDYQDYEPEA	143
tr|A0A093FVV3_DRYPU	PVDPENEAYEMPPEEDYQDYEPEA	143
tr|A0A087QQY8_APTFO	PVDPENEAYEMPPEEEYQDYEPEA	143
tr|R4GAF5_ANOCA	PVDPENEAYEMPPEEEYQDYEPEA	142
tr|A0A091I049_CALAN	AMDPENEAYEMPPEEEYQDYEPEA	143
tr|A0A091F433_CORBR	PVDPENEAYEMPPEEEYQDYEPEA	143
tr|A0A093PG66_PYGAD	PVDPENEAYEMPPEEEYQDYEPEA	143

```
tr|A0A091JLR4_EGRGA       PVDPENEAYEMPPEEEYQDYEPEA  143

tr|A0A091WCU5_OPIHO       PVDPENEAYEMPPEEEYQDYEPEA  143

tr|A0A091X556_NIPNI       PVDPENEAYEMPPEEEYQDYEPEA  143

tr|A0A093QA93_9PASS       PVDPENEAYEMPPEEEYQDYEPEA  143

tr|A0A0A0ANP9_CHAVO       PVDPENEAYEMPPEEEYQDYEPEA  143

tr|A0A093JQV1_STRCA       PVDPENEAYEMPPEEEYQDYEPEA  143

tr|A0A093CC60_TAUER       PMDPENEAYEMPPEEEYQDYEPEA  143

tr|Q9I9H1_CHICK           PVDPENEAYEMPPEEEYQDYEPEA  143

tr|G1N1K5_MELGA           PVDPENEAYEMPPEEEYQDYEPEA  143

tr|A0A2D4GM30_MICCO       PADPENEAYEMPPEEEYQDYEPEA  139

                                  ::*    *  **** ***
```

Fig. S10. Multiple sequence alignment (using Clustal Omega, https://www.ebi.ac.uk/Tools/msa/clustalo/) of human α-Syn with 80 other α-Syn from various species. The overall protein, and four Tyr residues are well conserved (highlighted in red), but C-terminal region (contains Y125, Y133, and Y136) show more variability compared to N-terminal (contains Y39).

Materials and Methods

General conditions

Lyophilized protein was dissolved in 20 mM MOPS pH 7.5, filtered through 0.22 µm filter (frisenette), and concentration determined based on absorbance at 280 nm (Nanodrop N1000, ThermoScientific) using a theoretical extinction coefficient of 0.412 $(mg/mL)^{-1}cm^{-1}$ for wt α-Syn, 4470 $M^{-1}cm^{-1}$ for Y39F and 1490 $M^{-1}cm^{-1}$ for Y125/133/136F. 100 mM stock solutions of H_2O_2 (Merck) was prepared fresh and concentration determined using an extinction coefficient of either 10 $M^{-1}cm^{-1}$ or 13 $M^{-1}cm^{-1}$ at 265 nm or 260 nm, respectively. A 60 mM $CuCl_2$ stock solution was used for MCO reactions.

Protein production

α-Syn was recombinantly expressed and purified as described [2]. Tyr→Phe mutants were generously provided by Prof. Hilal Lashuel. Mutant versions of α-Syn were expressed and purified according to the same protocol as for wt.

Oxidation of aSyn

α-Syn was oxidized at 37°C according to [3], with the ratios α-Syn:$CuCl_2$:H_2O_2 1:2.3:7.8 in all experiments unless stated otherwise. $CuCl_2$ was always added last. Oxidation was quenched by immobilized catalase beads (Biovision) and by addition of Na_2-EDTA at a final concentration of 2 mM.

Tyr fluorescence

Intrinsic Tyr and diTyr fluorescence were followed on a Cary Eclipse Fluorescence Spectrophotometer. Tyr fluorescence were measured at excitation 260 nm and emission at 305 nm. DiTyr fluorescence was measured by excitation at 320 nm and emission at 405 nm. Baseline of α-Syn alone were measured prior to addition of $CuCl_2$ and H_2O_2 to initiate diTyr formation. Kinetics were fitted to an exponential model.

ESI-MS analysis of intact protein

Protein samples were desalted using reversed-phase chromatography and subsequently on-line mass-analyzed by electrospray ionization (ESI) mass spectrometry (Synapt G2 HDMS mass spectrometer, Waters Corp., Milford, MA). The samples were desalted for 2 min on column (phenyl-based, MassPREP Micro Desalting Column, 2.1 × 5.0 mm, Waters) with a flow of 500 µL/min of 0.23% (v/v) aqueous formic acid provided by an Agilent 1260 Infinity quaternary pump (Agilent Technologies, Santa Clara, CA). Subsequently, the sample was eluted from the column with a short gradient (4 min) at a flow of 50 µL/min of 5% to 90% acetonitrile with 0.23 % (v/v) formic acid by a nanoAcquity UPLC Binary Solvent Manager (Waters) into the electrospray ion source. The desalting column was mounted in an HDX-Manager system (Waters). The ESI source was operated in positive ion mode with ESI spray voltage of +3.0 kV, sampling cone 20 V, extraction cone 3.6 V. Raw ESI-mass spectra were deconvoluted with the MaxEnt 1 Maximum Entropy algorithm in the MassLynx ver. 4.1 software package.

LC-MS/MS

Due to the absence of cysteines in αSN, the samples were not reduced and alkylated before digestion. The samples were digested with 2% chymotrypsin overnight at 37°C and afterwards lyophilized. The digested αSN was subjected to reversed-phase chromatography using an EASY nano-LC system (Thermo Fisher Scientific/Proxeon Biosystems). The samples were resuspended in 0.1% FA and loaded onto a

homemade 100 μm ID pre-column packed with ReproSil Pur C18 RP material (5 μm), and eluted from the pre-column onto an analytical 75 μm ID column also packed with ReproSil Pur C18 material (3 μm), using a gradient of 0-34% buffer B in 17 or 30 min and 34-100% B in 5 min and 100% buffer B in 8 min (Buffer A: 0.1% FA; Buffer B: 95% ACN/0.1% FA). Nano-electrospray ionization LC-MS/MS analysis was carried out using a Q Exactive HF Mass Spectrometer at a resolution of 120,000 in MS mode and 15,000 in MS/MS mode. The 10 most abundant peaks were chosen for MS/MS analysis and fragmented in HCD mode with normalized collision energy of 28. The MS raw files were processed and searched in Mascot through the Proteome Discoverer software (Thermo Fisher Scientific). Database searches were performed with the following parameters: Precursor mass tolerance of 10 parts per million (ppm); MS/MS mass tolerance of 0.05 Da.

Top-down sequencing

70 μM α-Syn samples (oxidized and non-oxidized) was buffer exchanged to 1 M Ammonium acetate using P6-biospin columns (BioRad), and diluted 5x in 1 M Ammonium Acetate prior to analysis. Intact native and oxidized α-Syn was analyzed on an Orbitrap Fusion Tribid mass spectrometer with an offline nanospray source using borosilicate capillaries (Thermo). Positive ionization mode with a capillary voltage of 1800 V was used. To exclude the formation of a dehydromethionine on Met1 upon oxidation top-down sequencing was performed. The 9+ state (1607 m/z) of the 1 min oxidized alpha-syn was fragmented using a reaction time with ETD reagent at 20 ms and a supplemental HCD activation (15 percent) was employed. The software, Excalibur, was used for processing of the data.

Immunoblotting

α-Syn (70 μM) was oxidized according to the protocol, and quenched at time points, 0 (unoxidized), 3, 15 and 60 min. All samples are mixed with non-reducing sample buffer, whereas only the oxidized α-casein sample (positive control) was further heated for 5 min at 95°C before 10 μL α-Syn sample was loaded on a 4-15% TGX gel from BioRad (6 μL α-casein). Pageruler plus prestained protein ladder, ThermoFisher Scientific was used as a marker. The gel was run in Laemmli buffer, and then transferred to a nitrocellulose membrane. After transfer, the blot was blocked in TBS-T (0.1%) containing 0.5 % BSA for 90 min on a rolling table at room temperature. The blot was washed four times in TBS-T before the primary antibody, anti-diTyr monoclonal antibody (Japan Institute for the Control of Aging; JaIC) is added 1:500 (100 μg/ml) in TBS-T/BSA. After overnight incubation at 4°C the blot was washed four times in TBS-T, and incubated for 1 h at room temperature with goat-anti mouse HRP (1:20000, v/v) before the images were developed.

SAXS

α-Syn samples were oxidized for 1 h as described. Oxidized monomers or untreated α-Syn monomers were purified on a Superose 6 10/300 increase column, in 20 mM MOPS, pH 7.5 prior to SAXS analysis. The samples were investigated by SAXS on the in-house Bruker AXS NanoSTAR instrument at Aarhus University, described in detail in [4]. The instrument uses an Excillum metal jet X-ray source and a homebuilt scatterless apertures in front of the sample (Pedersen, J.S. (Inventor) US patent number US9958404B2 (2018), EU number Patent EP13159569.6 (2019), Chinese Patent CN104132954B (2019), Japanese Patent JP6403964B2 (2018. X-ray analysis system for X-ray scattering analysis) and is equipped with an automated sample handler. Samples and buffer were measured in the same capillary and the buffer scattering was subtracted from that of the sample. The SAXS intensity, $I(q)$, which is a function of modulus of the scattering vector, q, was converted to absolute scale using the scattering from a pure water sample at 25 °C. The data were analyzed by Indirect Fourier Transformation [5, 6], which gives the pair distance distribution function, $p(r)$, which is a histogram of distance between pair of points weighted by the excess scattering length density at the points.

The data were also analyzed using polymer models. Models obeying Gaussian random-flight statistics, which neglects excluded volume effects are applicable, since the size of the chains relative to the local stiffness as measured by the Kuhn length is relatively small [7]. The data for the α-Syn native monomer was fitted by the function for a linear polymer [8]:

$$P_{chain}(q) = \frac{2(\exp(-x)-1+x)}{x^2} \quad \text{with } x = \frac{Lb}{6}q^2 \qquad (1)$$

where L is the contour length:

$$L = nl_0 \qquad (2)$$

where n is the number of residues (= 140 for α-Syn) and l_0 is the contour length of a peptide bond in the chain backbone (l_0 = 0.4 nm) [9] so that L = 56.0 nm. The parameter, b, is the Kuhn length, which describes the local stiffness of the chain. It was kept as a fit parameter when the expression was fitted to the SAXS data. The model was expressed on absolute scale so that:

$$I(q) = cM\Delta\rho_m^2 P_{chain}(q) + bck \qquad (3)$$

where c is the mass concentration, M = 14.4 kDa is the molecular mass, $\Delta\rho_m$ is the excess scattering length density per unit mass for protein, for which a typical value of 2.00×10^{10} cm/g, was used, and bck is a background. The parameter b, c and bck were used as fit parameters when the expression was fitted to the data using weighted least-squares methods [10]. Note that c can be compared to the value determined by absorbance measurements and that the Kuhn length b, can be compared to the value of b = 1.51 nm estimated from the expression given in [11] for unfolded proteins using the contour length calculated as described above.

For the oxidized monomer, we hypothesize that the Tyr residue Y39 cross links to one of the three Tyr residues in the C terminal (Y125, Y133, Y136), and that this leads to a conformation change with a loop or ring structure with contour length L_{loop}, with two linear chains attached to the ring, where one is relatively short with contour length L_{lin1} and the other one is longer with contour length L_{lin2} (see insert in Fig. 2). Using the formalism in [12], the normalized form factor can be calculated to be:

$$P(q) = \frac{1}{(L_{loop}+L_{lin1}+L_{lin2})^2}\left[L_{loop}^2\, P_{loop}(q) + L_{lin1}^2 P_{chain}(q, L_{lin1}) + L_{lin2}^2 P_{chain}(q, L_{lin2}) \right. \quad (4)$$

$$+2L_{loop}L_{lin1}\, A_{loop}(q)A_{chain}(q, L_{lin1}) + 2L_{loop}L_{lin2}\, A_{loop}(q)A_{chain}(q, L_{lin2}) \quad (5)$$

$$\left. +2L_{lin1}L_{lin2}\, A_{chain}(q, L_{lin1})A_{chain}(q, L_{lin2}) \right] \quad (6)$$

where

$$P_{loop}(q) = \frac{D[y]}{y}, \text{ with } y = q\sqrt{\frac{L_{loop}b}{24}} \quad (7)$$

is the form factor of the loop and $D[y]$ is the Dawson integral (Note that there is an error in the expression for $P_{loop}(q)$ in Svaneborg and Pedersen) [13]. For loops, $P_{loop}(q) = A_{loop}(q)$, where $A_{loop}(q)$ is the scattering amplitude of a loop. Furthermore,

$$A_{chain}(q, L) = \frac{1-\exp(-x)}{x} \quad (8)$$

is the scattering amplitude of a linear chain. For α-Syn, we estimate the contour length to: L_{loop} = 36.8 nm, L_{lin1} = 3.6 nm, L_{lin2} = 15.2 nm. The intensity expression used for fitting the data were expressed on absolute scale as described above for the native monomer model, and the concentration, Kuhn length, and background were fit parameters.

IM-MS

α-Syn wt, Y39F and Y125/133/136F were oxidized for 5 min before quenching the reaction. Ion mobility spectra are recorded on a SynaptG2S travelling wave ion mobility mass spectrometer equipped with an offline nanospray source (Waters, UK). nESI capillaries are purchased from Thermo. The capillary voltage was 1.5kV, the source temperature 100C, and the source pressure was 5.8 mbar. The IMS wave velocity was 800 m/s and the height was 30 V. CIU of native α-Syn was done in 10 V increments from 5 V to 45 V. Denatured myoglobin is used as calibrant.

Thioflavin T fluorescence

The amyloid specific fluorescent probe, Thioflavin T (ThT) was used to follow α-Syn amyloid formation. α-Syn (560 μM) was mixed with $CuCl_2$ and H_2O_2 in a 1:2.3:7.8 ratio, or controls with α-Syn alone or with either $CuCl_2$ or H_2O_2. Oxidized monomers were purified on a superose 6 10/300 increase column and identified on a 4-15% gel, TGX, BioRad, pooled and concentrated, if needed, using amicon 10 kDa cutoff concentration filters. ThT was added to a final concentration of 40 μM. Measurements were performed in a black 96-well clear bottom plate. A 3 mm glass bead (Sigma) was added for increased reproducibility [14].

Circular Dichroism Spectroscopy

Far-UV CD was used to characterize the secondary structure of endpoint α-Syn samples with or without oxidation. Spectra were recorded in the range 195 nm – 260 nm on a Chirascan-plus qCD spectrometer, Applied Photophysics, at room temperature (20°C) using a 1 mm cuvette with a protein concentration 14 μM. Background subtraction was made with the corresponding buffer (20 mM MOPS, pH 7.5). An average of three acquisitions was made. Units were converted to mean residue ellipticity for analysis.

Fourier-transformed infrared spectroscopy

Endpoint samples were loaded (2µL) on a quartz crystal on Tensor 27 FTIR, Bruker, Bruker Optics, Billerica, MA, USA and dried out using nitrogen gas. Spectra were obtained from accumulation of 68 scans in the range 1000 to 3998 cm^{-1} with 2 cm^{-1} resolution. The software, OPUS, was used for performing atmospheric compensation, baseline subtraction and second derivative analysis. All spectra were normalized.

Transmission Electron Microscopy

Endpoint α-Syn fibrillated samples were analyzed as described [15].

Bioinformatics analysis

For the analysis of Tyr residues in intrinsically disordered proteins (IDPs), protein disorder information for human proteome was obtained from MobiDB (http://mobidb.bio.unipd.it/). Closely similar sequences (>40% identity) were removed using H-CD-HIT (http://weizhongli-lab.org/cd-hit/) to get a set of non-redundant proteins (n=15015). Percentages of Tyr residues in disordered and ordered regions were computed using Python. Orthologs of human α-Syn were identified using BLAST and sequences were downloaded from UniProt (https://www.uniprot.org/). Multiple sequence alignment (MSA) was done using Clustal Omega (https://www.ebi.ac.uk/Tools/msa/clustalo/). α-Syn structure was obtained from PDB (https://www.rcsb.org/) and visualized using VMD (www.ks.uiuc.edu/Research/vmd/).

References

1. Khokhlov, A. R.; Grosberg, A. Y.; Pande, V. S., *Statistical Physics of Macromolecules*. American Institute of Physics Melville, NY: 1994; p 350.
2. Lorenzen, N.; Nielsen, S. B.; Buell, A. K.; Kaspersen, J. D.; Arosio, P.; Vad, B. S.; Paslawski, W.; Christiansen, G.; Valnickova-Hansen, Z.; Andreasen, M.; Enghild, J. J.; Pedersen, J. S.; Dobson, C. M.; Knowles, T. P. J.; Otzen, D. E., The Role of Stable alpha-Synuclein Oligomers in the Molecular Events Underlying Amyloid Formation. *Journal of the American Chemical Society* **2014,** *136* (10), 3859-3868.
3. Tiwari, M. K.; Leinisch, F.; Sahin, C.; Moller, I. M.; Otzen, D. E.; Davies, M. J.; Bjerrum, M. J., Early events in copper-ion catalyzed oxidation of alpha-synuclein. *Free Radical Bio Med* **2018,** *121,* 38-50.
4. Lyngso, J.; Pedersen, J. S., A high-flux automated laboratory small-angle X-ray scattering instrument optimized for solution scattering. *Journal of Applied Crystallography* **2021,** *54* (1), 295-305.
5. Glatter, O., New Method for Evaluation of Small-Angle Scattering Data. *Journal of Applied Crystallography* **1977,** *10* (Oct1), 415-421.
6. Pedersen, J. S.; Hansen, S.; Bauer, R., The aggregation behavior of zinc-free insulin studied by small-angle neutron scattering. *Eur Biophys J* **1994,** *22* (6), 379-89.
7. Pedersen, J. S.; Laso, M.; Schurtenberger, P., Monte Carlo study of excluded volume effects in wormlike micelles and semiflexible polymers. *Phys Rev E Stat Phys Plasmas Fluids Relat Interdiscip Topics* **1996,** *54* (6), R5917-R5920.
8. Debye, P., Molecular-weight determination by light scattering. *J Phys Colloid Chem* **1947,** *51* (1), 18-32.
9. Ainavarapu, S. R.; Brujic, J.; Huang, H. H.; Wiita, A. P.; Lu, H.; Li, L.; Walther, K. A.; Carrion-Vazquez, M.; Li, H.; Fernandez, J. M., Contour length and refolding rate of a small protein controlled by engineered disulfide bonds. *Biophys J* **2007,** *92* (1), 225-33.
10. Pedersen, J. S., Analysis of small-angle scattering data from colloids and polymer solutions: modeling and least-squares fitting. *Adv Colloid Interfac* **1997,** *70,* 171-210.
11. Kohn, J. E.; Millett, I. S.; Jacob, J.; Zagrovic, B.; Dillon, T. M.; Cingel, N.; Dothager, R. S.; Seifert, S.; Thiyagarajan, P.; Sosnick, T. R.; Hasan, M. Z.; Pande, V. S.; Ruczinski, I.; Doniach, S.; Plaxco, K. W., Random-coil behavior and the dimensions of chemically unfolded proteins (vol 101, pg 12491, 2004). *P Natl Acad Sci USA* **2005,** *102* (40), 14475-14475.
12. Svaneborg, C.; Pedersen, J. S., A formalism for scattering of complex composite structures. II. Distributed reference points. *J Chem Phys* **2012,** *136* (15).
13. Casassa, E. F., Some Statistical Properties of Flexible Ring Polymers. *J Polym Sci Part A* **1965,** *3* (2pa), 605-+.
14. Giehm, L.; Lorenzen, N.; Otzen, D. E., Assays for alpha-synuclein aggregation. *Methods* **2011,** *53* (3), 295-305.
15. Sahin, C.; Lorenzen, N.; Lemminger, L.; Christiansen, G.; Moller, I. M.; Vesterager, L. B.; Pedersen, L. O.; Fog, K.; Kallunki, P.; Otzen, D. E., Antibodies against the C-terminus of alpha-synuclein modulate its fibrillation. *Biophys Chem* **2017,** *220,* 34-41.
